# The Alzheimer susceptibility gene *BIN1* induces isoform-dependent neurotoxicity through early endosome defects

**DOI:** 10.1186/s40478-021-01285-5

**Published:** 2022-01-08

**Authors:** Erwan Lambert, Orthis Saha, Bruna Soares Landeira, Ana Raquel Melo de Farias, Xavier Hermant, Arnaud Carrier, Alexandre Pelletier, Johanna Gadaut, Lindsay Davoine, Cloé Dupont, Philippe Amouyel, Amélie Bonnefond, Frank Lafont, Farida Abdelfettah, Patrik Verstreken, Julien Chapuis, Nicolas Barois, Fabien Delahaye, Bart Dermaut, Jean-Charles Lambert, Marcos R. Costa, Pierre Dourlen

**Affiliations:** 1 Univ. Lille, Inserm, CHU Lille, Institut Pasteur Lille, U1167 - RID-AGE - Facteurs de risque et déterminants moléculaires des maladies liées au vieillissement, F-59000 Lille, France; 2 Brain Institute, Federal University of Rio Grande do Norte, Natal, Brazil; 3 Univ. Lille, Inserm, CNRS, CHU Lille, Institut Pasteur de Lille, U1283-UMR 8199 EGID, F-59000 Lille, France; 4 Univ. Lille, CNRS, Inserm, CHU Lille, Institut Pasteur de Lille, U1019-UMR 9017-CIIL-Center for Infection and Immunity of Lille, F-59000 Lille, France; 5 VIB Center for Brain and Disease Research, KU Leuven, Leuven, Belgium; 6 Department of Neurosciences, Leuven Brain Institute, KU Leuven, Leuven, Belgium; 7 Univ. Lille, CNRS, Inserm, CHU Lille, Institut Pasteur de Lille, US41-UMS2014-PLBS, F-59000 Lille, France; 8 Department of Biomolecular Medicine, Faculty of Medicine and Health sciences, Ghent University, 9000 Ghent, Belgium; 9 Centre for Medical Genetics, Ghent University Hospital, 9000 Ghent, Belgium

**Keywords:** BIN1 isoforms, Neurodegeneration, Early endosome, Alzheimer’s disease, Drosophila, Human induced neurons

## Abstract

The *Bridging Integrator 1* (*BIN1*) gene is a major susceptibility gene for Alzheimer’s disease (AD). Deciphering its pathophysiological role is challenging due to its numerous isoforms. Here we observed in Drosophila that human *BIN1* isoform1 (BIN1iso1) overexpression, contrary to human *BIN1* isoform8 (BIN1iso8) and human *BIN1* isoform9 (BIN1iso9), induced an accumulation of endosomal vesicles and neurodegeneration. Systematic search for endosome regulators able to prevent BIN1iso1-induced neurodegeneration indicated that a defect at the early endosome level is responsible for the neurodegeneration. In human induced neurons (hiNs) and cerebral organoids, *BIN1* knock-out resulted in the narrowing of early endosomes. This phenotype was rescued by BIN1iso1 but not BIN1iso9 expression. Finally, BIN1iso1 overexpression also led to an increase in the size of early endosomes and neurodegeneration in hiNs. Altogether, our data demonstrate that the AD susceptibility gene *BIN1*, and especially BIN1iso1, contributes to early-endosome size deregulation, which is an early pathophysiological hallmark of AD pathology.

**Supplementary Information:**

The online version contains supplementary material available at 10.1186/s40478-021-01285-5.

## Introduction

Alzheimer’s disease is the most common form of dementia, characterized by two main cerebral lesions: the extracellular aggregation of the amyloid beta peptide (Aβ) into senile plaques and the intracellular aggregation of phosphorylated Tau into tangles. In addition, other cytopathological features specific to familial and sporadic AD can be also observed such as abnormally enlarged early endosomes in neurons [8]. At the genetic level, familial AD is due to mutations in *APP*, *PSEN1* and *PSEN2*. Sporadic AD is a multifactorial disease exhibiting a strong genetic component with an estimated attributable risk of 60–80% [20]. Over the last decade, our understanding of this genetic component has strongly progressed with the identification of 76 loci associated with the disease [4]. Among these loci, *BIN1* is the second AD susceptibility gene after *APOE* in terms of association [4, 33, 35].

*BIN1* encodes at least 20 exons subject to extensive differential splicing, generating multiple isoforms with different tissue distributions [49]. *BIN1* isoform1 (BIN1iso1) and *BIN1* isoform8 (BIN1iso8) are respectively expressed in the brain and skeletal muscles, the two tissues where *BIN1* is mostly expressed, whereas *BIN1* isoform9 (BIN1iso9) is ubiquitously expressed (GTEx portal, http://www.gtexportal.org). In the brain, BIN1iso1 and BIN1iso9 are the most abundant isoforms [11, 61]. All *BIN1* isoforms possess the N terminal BIN1/Amphiphysin/Rvs (BAR) domain, involved in membrane curvature sensing and induction, the C-terminal MYC-Binding Domain (MBD) and the C-terminal SH3 domain, a protein–protein interaction domain that recognizes proline-rich domains like the one in Tau [49, 59]. Muscle-specific isoforms contain a phosphoinositide-interacting (PI) domain, whereas brain-specific *BIN1* isoforms are mainly characterized by inclusion of exons encoding a Clathrin and Adaptor Protein-2 binding (CLAP) domain involved in endocytosis and intracellular trafficking. In the brain, a complex expression pattern is also observed at the cellular level. *BIN1* expression is mainly observed in oligodendrocytes, microglial cells and neurons [1, 41, 52]. However, while neurons express high molecular weight isoforms including BIN1iso1, glial cells express lower molecular weight isoforms such as BIN1iso9 [52, 69].

AD-associated *BIN1* variants are non-coding and likely regulate *BIN1* expression [9]. However, the dysregulation of *BIN1* expression in the brain of AD cases is still highly debated. Some results indicate that overall *BIN1* expression is increased [9], or decreased [21, 41], whereas more complex patterns have been also reported with a decrease in BIN1iso1 and a concomitant increase in BIN1iso9 expression [24]. In addition, according to the pattern of expression, it is not clear if the observed variations of *BIN1* expression are a cause or a consequence of the neurodegenerative process. For example, the decrease in BIN1iso1 and increase in BIN1iso9 expressions may be a consequence of neuronal death and gliosis, respectively, as *BIN1* isoform variations are correlated with neuronal and glial marker variations [52]. Therefore, based on its global and/or isoform expression variation, it is difficult to assess whether BIN1 may be deleterious or protective in AD.

Importantly, impact of such global and/or isoform expression deregulations on the AD pathophysiological process has not yet been elucidated even if several hypotheses have been proposed: (i) modulation of Tau function and neurotoxicity though interaction of the BIN1 SH3 domain with the Tau proline-rich domain in a phosphorylation-dependent manner [39, 54, 59]; (ii) modulation of Tau spreading through its role in endocytosis and intracellular trafficking or extracellular vesicles [6, 11]; (iii) regulation of the APP metabolism despite contradictory results in different models [2, 62]; (iv) regulation of synaptic transmission either in the presynaptic [53] or postsynaptic compartment [56].

Within this complex background, considering the numerous *BIN1* isoforms and the different functions regulated by this gene, it is, thus, pivotal to address isoform-specific functions of BIN1 towards a comprehensive understanding of its role in AD pathophysiology. For this purpose, we investigated the role and potential toxicity of *BIN1* isoforms in neuronal cells by focusing on BIN1iso1, BIN1iso8 and BIN1iso9. We used the highly tractable and readout-rich Drosophila model which allowed the assessment of *BIN1* isoform neurotoxicity in vivo during aging. We further analyzed the role of *BIN1* isoforms in hiNs derived from human induced pluripotent stem cells (hiPSC), a model closer to AD pathology.

## Materials and methods

### Drosophila genetics and behavioral experiments

Flies were raised at 25 °C under a light/dark cycle of 12 h/12 h (3000 lx) on standard fly medium (Nutri-fly MF, Genesee Scientific, San Diego, CA, USA), unless otherwise stated. UAS-BIN1iso1, UAS-BIN1iso1 ΔEx7, UAS-BIN1iso1 ΔCLAP, UAS-BIN1iso9, UAS-BIN1iso8 and UAS-dAmphA lines were generated in this work (see Additional file 1). Briefly, cDNA were subcloned into pUASTattB vector and injected in attP2 lines (on the III chromosome) and in attP40 lines (on the II chromosome) (BestGene Inc., CA, USA). rh1-Gal4, GMR-Gal4, rh1-GFP, UAS-GFP:ninaC, UAS-evi:GFP, UAS-GFP:LC3 were described previously [13, 17, 36, 40]. The Amph^5E3^ line was a kind gift from GL Boulianne [37]. Other stocks were obtained from the Bloomington Drosophila Stock Center (BDSC, Bloomington, IN, USA): UAS-Luciferase (#35788), UAS-mCD8:GFP (#27400), UAS-GFP (#35786), Amph^MI08903-TG4.0^ (#77794), Rab5^EYFP^ (#62543), Rab7^EYFP^ (#62545), UAS-GFP-myc-2xFYVE (#42712), UAS-GFP-Rab5 (#43336), UAS-YFP.Rab5.Q88L (#9771), UAS-YFP.Rab5.S43N (#9774), Rab5^[2]^ (#42702), P{TRiP.HMS00147}attP2 Rab5 (#34832), UAS-Rab7.GFP (#42705), UASp-YFP.Rab7.Q67L (#24103), UASp-YFP.Rab7.T22N (#9778), UAS-Rab11-GFP (#8506), UASp-YFP.Rab11.Q70L (#9791), UASp-YFP.Rab11.S25N (#9792), P{TRiP.HMS01056}attP2 Vha68-2 (#64582), P{TRiP.HMS01442}attP2 VhaAC39-1 (#35029), UAS-mCherry:NLS (#38424), attP2 empty line (#8622), UASp-YFP.Rab9 (#9784), Rab1^EYFP^ (#62539), Rab6^EYFP^ (#62544), UAS-GFP.KDEL (#9898), UAS-ManII-EGFP (#65248), UAS-GFP-LAMP (#42714), Appl[d] (#43632), UAS-Appl (#38403), UAS-APP695 (#33796), UAS-YFP:Rab4 (#23269), UAS-YFP:Rab4[S22N] (#9768), UAS-YFP:Rab4[Q67L] (#9770), UAS-Rab4:mRFP (#8505) and UAS-mCD8:mRFP (#27400).

For the climbing test, 5 flies were subjected together to testing in a graduated cylinder. The wall of the cylinder had 5 main graduations, the top one being 13 cm from the bottom. Flies were tapped down to the bottom of the cylinder and recorded for 10 s to see if they climbed up the wall of the cylinder. This was repeated 5 times in total. A score corresponding to the distance they had climbed was determined by the recorded movies. Flies got a score of between 0 and 5 depending on the main graduation that they were able to reach during the 10 s period. The mean of the 5 trials was calculated and attributed to each fly.

### Western blot of Drosophila samples

Drosophila heads (n = 10) and thorax (n = 5) were dissected and crushed in ice-cold LDS lysis buffer (NP0008, NuPAGE, Novex, Life Technologies) supplemented with reducing agent (NP0009, NuPAGE, Novex, Life Technologies). Samples were centrifuged at 8500*g* for 10 min at 4 °C. Supernatants were kept at − 80 °C. Once thawed, they were boiled for 10 min at 85 °C before being loaded and separated in SDS–polyacrylamide gels 4–12% (NuPAGE Bis–Tris, ThermoScientific) in MOPS 1X buffer (NP0001-02, NuPAGE, Novex, Life Technologies). After migration, samples were transferred on to nitrocellulose membranes using the Biorad Trans-blot transfert system kit (Biorad) according to the supplier technical recommendation (7 min, 2.5 A, 25 V). Next, membranes were incubated in milk (5% in Tris-buffered saline with 0.1% Tween-20) to block non-specific binding sites during 1 h at RT, followed by several washes. Immunoblotting was carried out with primary antibodies anti-BIN1 (BIN1 99D, 05-449, Millipore, RRID:AB_309738, 1/2500; BIN1 ab27796, abcam, RRID:AB_725699, 1/1000), anti-α-tubuline (α-tubuline DM1A, T9026, Sigma, RRID:AB_477593, 1/5000), anti-dAmph (#9906, kind gift of Andrew Zelhof, 1/5000)[67] and anti-GFP (anti-GFP, G1544, Sigma, RRID:AB_439690, 1/4000) overnight at 4 °C. After washing, membranes were incubated with HRP-conjugated secondary antibodies (Jackson, anti-mouse 115-035-003, RRID:AB_10015289 and anti-rabbit 111-035-003, RRID:AB_2313567) 2 h at room temperature. Immunoreactivity was revealed using the ECL chemiluminescence system (WBLUC0500, Immobilon Classico Western HRP Substrate, Millipore) and imaged using the Amersham Imager 600 (GE LifeSciences). Optical densities of bands were quantified using Fiji software and results were normalized with respect to tubulin expression [55].

### Cornea neutralization

CO2-anesthetized flies were placed in a 35 mm cell culture dish half-filled with 1% agarose and covered with water at 4 °C as described [14]. Flies were observed using an upright confocal microscope (Zeiss LSM710, Wetzlar, Germany) equipped with a 40 × water immersion long-distance objective. Images were acquired using the Zen acquisition software (Zeiss Zen software). Photoreceptor neurons were manually quantified.

### Immunofluorescence of Drosophila samples

Fly heads were dissected and fixed in 4% paraformaldehyde phosphate buffer saline (PBS) for 20 min at room temperature. After washing, retinas were finely dissected, permeabilized and depigmented in 0.3% (v/v) Triton X-100 in PBS (0.3% PBT) overnight at 4 °C under gentle agitation. After blocking with 5% normal goat/donkey serum in 0.3% PBT, samples were incubated overnight at 4 °C with the primary antibodies diluted in 0.3% PBT. The following antibodies were used: anti-NA/K ATPase alpha subunit (a5, DSHB, RRID:AB_2166869, 1/100), anti-rhodopsin (4C5, DSHB, RRID:AB_528451, 1/200) and anti-GFP (132004, Synaptic System, RRID:AB_11041999, 1/100). After washing, they were incubated overnight at 4 °C with Alexa 555 Phalloidin anti-F-actin (A34055, ThermoFisher Scientific) and the secondary antibodies diluted in 0.3% PBT: Alexa 488 Donkey anti-guinea pig (706-545-148, RRID:AB_2340472, Jackson ImmunoResearch), Alexa 633 Goat anti-mouse (A-21052, RRID:AB_2535719, ThermoFisher Scientific). After washing, samples were incubated in 90% glycerol PBS for 30 min in the dark before being mounted in the same solution. Retinas were imaged with a LSM710 confocal microscope (Zeiss, Wetzlar, Germany) equipped with a 40X oil objective.

### Electron microscopy

Drosophila eyes were dissected and fixed in 1% glutaraldehyde, 4% paraformaldehyde, 0.1 M sodium cacodylate buffer (pH 6.8) 30 min at room temperature and then overnight at 4 °C. After washing, eyes were post-fixed at room temperature in 1% OsO4 and 1.5% potassium ferricyanide for 1 h, then with 1% uranyl acetate for 45 min, both in distilled water at room temperature in the dark. After washing, they were dehydrated with successive ethanol solutions. Eyes were infiltrated with epoxy resin (EMbed 812 from EMS) and were mounted in resin into silicone embedding molds. Polymerization was performed at 60 °C for 2 days. Ultrathin sections of 70–80 nm thickness were observed on formvar-coated grid with a Hitachi H7500 TEM (Milexia, France), and images were acquired with a 1 Mpixel digital camera from AMT (Milexia, France).

### Maintenance of cells and generation of hiPSCs and neural derivatives

hiPSCs (ASE 9109, Applied StemCell Inc. CA, USA) modified for BIN1 in exon 3 (Fig. 5) were generated by CRISPR/Cas9. Homozygous null mutants for BIN1 had a 5 bp deletion on one allele and an 8 bp deletion on the other allele. All hiPSCs, and all subsequent human induced neural progenitor cells (hiNPCs), hiNs, human induced astrocytes (hiAs), and cerebral organoids derived thereof, were maintained in media from Stemcell Technologies, Vancouver, Canada. Maintenance of cell cultures and organoids were done in adherence with manufacturer’s protocols which can be found on the webpage of Stemcell Technologies. hiPSCs were maintained in mTeSR1 medium in non-treated cell culture dishes/plates pre-coated with vitronectin. Cell numbers and viability were recorded using a LUNA™ Automated Cell Counter.

In order to obtain hiNPCs, the embryoid body method detailed by Stemcell Technologies was used for the induction of BIN1 WT and KO hiPSCs. Following the generation of hiNPCs, these derived cells were maintained in treated cell culture dishes pre-coated with poly-L-ornithine (PLO) and laminin (5 µg/mL). PLO solution was made in water (0.001%) while laminin was diluted in PBS with Ca^2+^ and Mg^2+^. BIN1 WT and KO hiNPCs, thus generated, were maintained for up to 10 passages.

2D cultures comprising hiNs and hiAs were produced from hiNPCs. 60,000 hiNPCs/well were plated in 24-well cell imaging plates from Eppendorf (Cat # 0030741005) pre-coated with PLO (0.001%) and laminin (10 µg/mL). Cells were kept in 0.5 mL of NPC medium per well for 24 h. Following this, equal volume of complete BrainPhys medium (supplemented with BDNF, GDNF, laminin, dibutyryl-cAMP, ascorbic acid, N2, and SM1) was added to each well to begin the process of differentiation. Subsequently, media was changed in the plates bi-weekly. The media change consisted of removing half of the existing medium in each well and replacing it with an equal volume of fresh complete BrainPhys medium. Mixed cultures of hiNs and hiAs were obtained at the end of 6 weeks from the start of the differentiation process.

### Generation of cerebral organoids

Cerebral Organoids were generated from hiPSCs at 80% confluency. hiPSCs were detached from the Vitronectin XF substrate using Gentle Cell Dissociation Reagent (Stemcell Technologies), centrifuged, pelleted and resuspended in Embryoid Body (EB) seeding medium (Stemcell Technologies) to form EBs. 9000 cells were plated per well in a 96-well round-bottom ultra-low attachment plate. After two days, 1 or 2 EBs were transferred to a well of a 24-well ultra-low attachment plate containing Induction Medium (Stemcell Technologies). The EBs were kept in the induction Medium for 2 days and next they were transferred into Matrigel (Corning) using an embedding surface. When the Matrigel polymerized, the EB were transferred to a 6-well ultra-low adherent plate with Expansion Medium (Stemcell Technologies). After 3 days, the medium was replaced by Maturation Medium (Stemcell Technologies) and the plate was placed in an orbital shaker (65 rpm speed). Complete media changes were done on a bi-weekly basis.

### Lentiviral infections

Lentiviral constructs were produced by the Vect’UB platform within the TBM Core unit at University of Bordeaux, Bordeaux, France (CNRS UMS 3427, INSERM US 005). All three lentiviral constructs harbored reporter tags for the expression of tdTomato protein. The lentiviral vectors used were the empty vector—436 (ID # 1770), 436-Bin1Iso1 (ID # 1771), and 436-Bin1Iso9 (ID # 1772). Lentiviral infections were done in 3-week old differentiation cultures obtained from hiNPCs. Viral transductions were performed at a multiplicity of infection (MOI) of 1. In brief, appropriate volumes of each construct were mixed in complete BrainPhys medium and 50 ul of the viral medium mix was then added to each well. Each of the 3 constructs were added in triplicate to respective wells for each of the BIN1 WT and KO cells. Infected cells were maintained for a further 3-week period with bi-weekly changes of half volume of medium in each well.

### Immunocytochemistry of 2D cultures

Cells were fixed in PFA (4% w/v) for 10 min. Fixed cells were then washed with PBS 0.1 M. Cells were then blocked with blocking solution (5% normal donkey serum + 0.1% Triton X-100 in PBS 0.1 M) at room temperature for 1 h under shaking conditions. Primary antibodies diluted in the blocking solution were then added and incubation was done overnight at 4ºC under shaking conditions. The following day, cells were washed with PBS 0.1 M 3 times for 10 min each before the addition of fluorophore-conjugated secondary antibodies in blocking solution for 2 h at room temperature under shaking conditions ensuring protection from light. 3 washes with PBS were done for 10 min each at room temperature under shaking conditions with protection from light. Hoechst 33258 nucleic acid stain was added to PBS 0.1 M in the second wash. Cells were mounted with fluoromount and imaged directly in the cell imaging plates. All images were acquired using an LSM 880 Confocal Scanning Microscope housed at the Imaging Platform of the Institut Pasteur de Lille using the ZEISS ZEN Imaging Software. Image acquisition was done at 40X for the various cellular markers in Fig. 5. For EEA1 quantifications, we selected 10 random regions positive for MAP2 in 2–3 different wells from 3 independent cell culture batches. Images were taken using a 63X objective and zoom of 2.

### Immunohistochemistry of organoid samples

Cerebral organoids were fixed in PFA (4% w/v) for 30 min at 4 °C followed by three washes with PBS 0.1 M. Cerebral organoids were then placed in sucrose solution (30% w/v) overnight before being embedded in O.C.T (Tissue-Tek). Embedded tissue was sectioned at 20 μm using a cryostat and mounted slides were stored at − 80 °C until immunostaining was performed. Mounted tissue was removed from storage and warmed by placing at room temperature for 30 min. Tissue were rehydrated and washed with room temperature PBS 0.1 M 3 times for 5 min. Slides were then washed once with PBS with 0.2% Triton X-100 for 15 min. Tissue was blocked using 10% of donkey serum in PBS 0.1 M for 1 h at room temperature. After blocking, primary antibodies were added to 0.2% Triton X-100 and 10% of donkey serum in PBS 0.1 M at appropriate dilutions and incubated overnight at 4 °C. The next day, slides were washed with PBS 0.1 M 3 times for 5 min each with gentle shaking. Subsequently, slides were incubated with fluorophore-conjugated secondary antibodies in 0.2% Triton X-100 and 10% of donkey serum in PBS 0.1 M for 2 h at room temperature in the dark. After secondary antibody incubation, slides were washed 3 times with PBS for 5 min with gentle shanking. Nuclei were visualized by incubating the tissue for 5 min with Hoechst stain in PBS 0.1 M. Sections were mounted using aqueous mounting medium (Polysciences). Images were acquired using an LSM 880 Confocal Scanning Microscope in concert with the ZEISS ZEN imaging software housed at the Imaging Platform of the Pasteur Institute, Lille. For EEA1 quantifications, we selected 5 random regions in the surface (50-250 µm) of each cerebral organoid, where a high density of MAP2-positive cells could be distinguished. Images were acquired using a 63X objective in 2–3 different sections obtained from at least 3 cerebral organoids of each genotype.

### Antibodies used for immunocytochemistry/immunohistochemistry of hiNs and organoid samples

hiPSCs were detected with antibodies for SOX2 (RRID:AB_2651000) and SSEA4 (RRID:AB_2651001) using the Molecular Probes™ Pluripotent Stem Cell 4-Marker Immunocytochemistry Kit (Thermo Fisher Scientific, RRID:AB_2651000). Antibodies used for immunocytochemistry/immunohistochemistry were EEA1 (610457, BD Biosciences, RRID:AB_397830), MAP2 (188006, Synaptic Systems, RRID:AB_2619881), RFP (600-401-379, Rockland Immunochemicals, Inc., RRID:AB_2209751), SOX2 (14-9811-82, Invitrogen, RRID:AB_11219471), NESTIN (MAB5326, Millipore, RRID:AB_2251134), and GFAP (AB5804, Millipore, RRID:AB_2109645). All fluorophore-tagged secondary antibodies were sourced from Jacskon ImmunoResearch Europe Ltd.

### Immunoblotting of 2D cultures and cerebral organoid

Samples from the 2D cultures or brain organoids were collected in RIPA buffer containing protease inhibitors (Complete mini, Roche Applied Science, Penzberg, Germany) and sonicated two times at 60–70% during 10 s before use for the western blotting analyses.

Protein quantification was performed using the BCA protein assay (Thermo Scientific). In total, 10 μg of protein from extracts were separated in SDS–polyacrylamide gels 4–12% (NuPAGE Bis–Tris, Thermo Scientific) and transferred to nitrocellulose membranes (Bio-Rad). Next, membranes were incubated in milk (5% in Tris-buffered saline with 0.1% Tween-20 – TTBS, or SuperBlock – Thermo Scientific) to block non-specific binding sites during 1 h at RT, followed by several washes with TTBS. Immunoblottings were carried out with primary antibodies anti-BIN1 (ab182562, Abcam, RRID:AB_725699), anti-APP (C-terminal) (A8717, Sigma-Aldrich, RRID:AB_258409) and anti-β-ACTIN (A1978, Sigma-Aldrich, RRID:AB_476692) overnight at 4 °C on 20 RPM agitation. The membranes were washed three times in TTBS, followed by incubation with HRP-conjugated secondary antibodies anti-mouse (115-035-003, RRID:AB_10015289) and anti-rabbit (111-035-003, RRID:AB_2313567, Jackson ImmunoChemicals, Inc.) overnight at 4 °C on 20 rpm. The membranes were washed three times in TTBS, and the immunoreactivity was revealed using the ECL chemiluminescence system (SuperSignal, Thermo Scientific) and imaged using the Amersham Imager 600 (GE Life Sciences). Optical densities of bands were quantified using the Gel Analyzer plugin in Fiji – ImageJ [55].

### Image analysis using Imaris

The “surface” function on Imaris was used for detection of EEA1 puncta. Background subtraction was performed using “local contrast” and automatic threshold, warranting the same processing criteria for all images. Next, a manual filter cut-off was applied to detect all puncta volumes above 0.1 µm^3^. MAP2 and tdTomato surfaces were also detected based on background subtraction using “local contrast” and automatic threshold. Automatic thresholds using the filter “absolute intensity” were then applied to find MAP2 and tdTomato surfaces. For the detection of EEA1 puncta on MAP2 and EEA1 puncta on MAP2^+^ /tdTomato^+^ cells, filters for the standard deviation of intensity were applied in the MAP2 and tdTomato channels respectively. Volume information for the EEA1 puncta were collated from each acquired image as CSV files. The volumes were sorted using Microsoft Excel. We used a cut-off maximum volume of 10µm^3^ for each field. The sorted volume data were then analyzed statistically using the GraphPad Prism software.

### snRNA-seq library preparation

Nuclei isolation and Hash-tagging with oligonucleotides steps were realized on ice with pre-cold buffers and centrifugations at 4 °C. BIN1 WT and KO organoids of 6 months (n=4 per genotype) were cut in 2 parts, washed twice with 1 ml of Deionized Phosphate Buffer Saline 1X (DPBS, GIBCO™, Fisher Scientific 11590476) and centrifuged 5 min at 300*g*. Organoids pellets were resuspended in 500 μl lysis buffer (Tris–HCL 10 mM, NaCl 10 mM, MgCl_2_ 3 mM, Tween-20 0.1%, Nonidet P40 Substitute 0.1%, Digitonin 0.01%, BSA 1%, Invitrogen™ RNAseout™ recombinant ribonuclease inhibitor 0.04 U/μL). Multiple mechanical resuspensions and wrecking steps in this buffer were perform for a total lysis time of 10 min, 500 μl of washing buffer was added (Tris–HCL 10 mM, NaCl 10 mM, MgCl_2_ 3 mM, Tween-20 0.1%, BSA 1%, Invitrogen™ RNAseout™ recombinant ribonuclease inhibitor 0.04 U/μL) and the lysis suspension was centrifuged 8 min at 500*g* (used for all following centrifugation steps). Nuclei pellets were washed three times with one filtration step by MACS pre-separation filter 20 μm (Miltenyi Biotec). Nuclei pellets were resuspended in 100 μL of staining buffer (DPBS BSA 2%, Tween-20 0.01%), 10 μL of Fc blocking reagent HumanTruStainFc™ (422302, Biolegend) and incubated 5 min at 4 °C. 1 μl of antibody was added (Total-Seq™-A0451 anti-Vertebrate Nuclear Hashtag 1 MAb414 for the WT and Total-Seq™-A0453 anti-Vertebrate Nuclear Hashtag 3 MAb414 10 µg for the KO, 97284 and 97286 respectively, Biolegend) and incubated 15 min at 4 °C. Nuclei pellets were washed three times in staining buffer with one filtration step by MACS pre-separation filter 20 μm (Miltenyi Biotec) to a final resuspension in 300 μl of staining buffer for Malassez cell counting with Trypan blue counterstaining (Trypan Blue solution, 11538886, Fisherscientific). Isolated nuclei were loaded on a Chromium 10 × Genomics controller following the manufacturer protocol using the chromium single-cell v3 chemistry and single indexing and the adapted protocol by Biolegend for the HTO library preparation. The resulting libraries were pooled at equimolar proportions with a 9 for 1 ratio for Gene expression library and HTO library respectively. Finally, the pool was sequenced using 100 bp paired-end reads on the Illumina NovaSeq 6000 system following the manufacturer recommendations.

### snRNA-seq analysis and differential expression analysis

UMI Count Matrices for gene expression and for HTO libraries were generated using the CellRanger software (10 × Genomics). After filtering for low quality cells according to the number of RNA, genes detected, and percentage of mitochondrial RNA, and normalizing the HTO matrix using centered log-ratio (CLR) transformation, 2,990 cells were assigned back to their sample of origin using HTODemux function of the SeuratV3 R Package (Satijalab), resulting to 1,794 and 1,196 cells for BIN1 KO and WT, respectively. Then, Seurat Workflow with SCTransform normalization was used to cluster the cells according to their transcriptome similarities. Each cluster was annotated using cell type specific markers. Finally, differential expression analysis between BIN1 KO and WT cells within each identified cell type was performed using DESeq2 package [38].

### Statistical analyses

Statistical information are available in the method section and in the figure legends. Two-tailed statistical tests were used. Hypothesis testing was carried out with Kruskal Wallis test followed by Mann–Whitney comparison and ANOVA with post-hoc Tukey. Statistical analyses were performed using R 3.6.0 (R Core Team (2019). R: A language and environment for statistical computing. R Foundation for Statistical Computing, Vienna, Austria. URL https://www.R-project.org/), RStudio 1.2.1335 and GraphPad Prism. For boxplots, the bold segment, lower and upper hinges represent the median, first quartile and third quartile respectively. The upper whisker extends from the hinge to the largest value no further than 1.5 * IQR from the hinge (where IQR is the inter-quartile range, or distance between the first and third quartiles). The lower whisker extends from the hinge to the smallest value at most 1.5 * IQR of the hinge.

## Results

### Functional conservation of human BIN1 isoforms in Drosophila

**Fig. 1:**
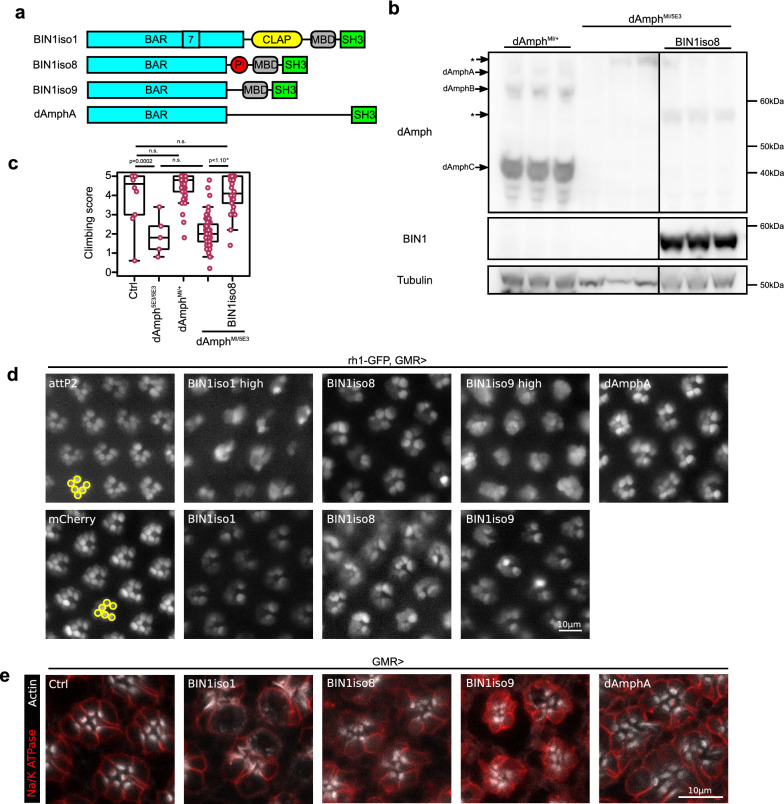
Functional conservation of human BIN1 isoforms in Drosophila. **a** Scheme of cerebral human BIN1 isoform1 (BIN1iso1), muscular human BIN1 isoform8 (BIN1iso8), ubiquituous human BIN1 isoform9 (BIN1iso9) and Drosophila BIN1, called Amphiphysin, isoformA (dAmphA) for which transgenic lines were generated. **b** Western blot analysis of Amph and BIN1 expression in dAmph^MI08903-TG4.0/+^, dAmph^MI08903-TG4.0/5E3^ and dAmph^MI08903-TG4.0/+^; UAS-BIN1iso8 fly thorax. dAmphA, dAmphB and dAmphC were expressed in the heterozygous dAmph^MI08903/+^ flies, whereas they were not detected in dAmph^MI08903/5E3^ flies (*background staining). BIN1iso8 was expressed in dAmph^MI08903-TG4.0/+^; UAS-BIN1iso8 flies. **c** Analysis of the climbing locomotor activity of 2 day-old flies with the indicated genotype. dAmph^MI08903-TG4.0/5E3^ flies exhibited a similar low climbing score as the null dAmph^5E3/5E3^flies. Expression of BIN1iso8 rescued the locomotor defects of dAmph^MI08903-TG4.0/5E3^ flies (from left to right n = 10, 5, 49, 67, 26, ANOVA F-value = 66.15, Df = 4, p = 2.37 × 10^−38^ with post-hoc Tukey, n.s. not significant). **d** Visualization of outer photoreceptor neuron rhabdomeres by cornea neutralization in 2-day-old flies expressing mCherry (as a control), BIN1iso1, BIN1iso8, BIN1iso9 and dAmphA under a GMR driver (attP2 is a control with an empty attP2 landing site and no UAS construct). While each ommatidium contained 6 outer photorceptors organized in a trapezoid shape (yellow circles) in the two control conditions, BIN1 isoforms and dAmphA expression resulted in a strong alteration in the number, shape and trapezoid organization of the rhabdomeres with a stronger effect for BIN1iso1 and BIN1iso9 high-expressing lines. **e** Immunofluorescence of whole-mount pupal retina expressing Luciferase (as a control), BIN1iso1, BIN1iso8, BIN1iso9 or dAmphA. They were labelled for the plasma membrane neuronal Na/K ATPase and F-actin. Contrary to the control, BIN1iso1, BIN1iso8, BIN1iso9 and dAmphA induced a strong accumulation of F-actin at the level of the rhabdomere

*BIN1* belongs to the amphiphysin family and in mammals, two genes compose this family: *amphiphysin I* (*AMPH*) and *amphiphysin II* (*AMPH2*) also named *BIN1*. Drosophila has only one ortholog called *Amphiphysin* (*Amph*) that is referred to as *dAmph* henceforth in this article. dAmph has 3 isoforms but only the BAR and SH3 domains are conserved in dAmph. Within this background, we generated 3 transgenic Drosophila lines expressing the human BIN1iso1, BIN1iso8 and BIN1iso9 isoforms. As a control, we also generated transgenic Drosophila lines expressing the longest dAmph isoform, dAmphA (Fig. 1a). We obtained transgenic lines expressing identical basal level of BIN1 isoforms with two additional BIN1iso1 and BIN1iso9 lines expressing high BIN1 levels that we used for dose-dependent effects (Additional file 1 and Additional file 2: Fig. S1).

We assessed the functional conservation of human BIN1 isoforms in Drosophila. Like human subjects harboring *BIN1* coding mutations and suffering myopathy [44], *dAmph* null flies have locomotor defects, due to T-tubule morphogenesis defects in muscle cells [37, 67]. We tested if the expression of muscle human BIN1iso8 could restore the locomotor performance of *dAmph* deficient adult transgenic flies assessed in the so-called climbing test. In addition to the null dAmph^5E3^ allele, we took advantage of a dAmph^MI08903-TG4.0^ allele that allows Gal4 expression under the control of *dAmph* endogenous promoter while stopping *dAmph* transcription (Additional file 1 and Additional file 3: Fig. S2). We checked by western blot that *dAmph* expression was abolished in dAmph^MI08903-TG4.0/5E3^ compound heterozygous flies compared to dAmph^MI08903-TG4.0/+^ heterozygous flies and that BIN1iso8 could be expressed in this genetic background (Fig. 1b). Then, we observed that dAmph^MI08903-TG4.0/5E3^ compound heterozygous flies had locomotor defects like dAmph^5E3/5E3^ null flies compared to control flies or dAmph^MI08903-TG4.0/+^ heterozygous flies (Fig. 1c). Expression of human BIN1iso8 restored the locomotor abilities of dAmph^MI08903-TG4.0/5E3^ compound heterozygous flies to levels similar to the ones observed in control flies or dAmph^MI08903-TG4.0/+^ heterozygous flies (Fig. 1c). Thus, human BIN1iso8 is able to rescue the locomotor functions of *dAmph* null flies, thereby indicating a functional conservation of human BIN1iso8 in Drosophila.

Next, we addressed the possible functional conservation of BIN1 isoforms in neuronal cells. Overexpression of dAmphA results in development defects of the light-sensitive photoreceptor neurons in Drosophila retina [67]. These neurons possess a specialized compartment, called rhabdomere, which consists of an apical microvillar stack of actin-rich intricately folded membranes containing the light-sensing rhodopsin proteins. We tested if human BIN1 isoforms expression with the early eye-specific GMR driver could phenocopy the dAmphA-induced rhabdomere phenotype. The Drosophila eye is composed of 600–800 units called ommatidia. Each ommatidium contains 6 outer photoreceptor neurons and 2 superimposed inner photoreceptor neurons. We expressed GFP in the outer photoreceptor neurons (Rh1 driver) and used the cornea neutralization technique to assess rhabdomere morphology [13]. While we observed six rhabdomeres per ommatidium, of similar size and organized in a trapezoidal shape in the control condition, some rhabdomeres were missing and others were smaller or deformed in the dAmphA overexpressing condition (Fig. 1d). Thus, we confirmed that expression of dAmphA alters rhabdomere morphogenesis. Individual expression of BIN1iso1, BIN1iso8 or BIN1iso9 recapitulated a similar phenotype respectively (Fig. 1d). In addition, high levels of BIN1iso1 and BIN1iso9 exacerbated the phenotype indicating a dose-dependent effect. We also confirmed these results on whole-mount pupal retina dissection (Fig. 1e). dAmphA-, BIN1iso1-, BIN1iso8- and BIN1iso9- overexpressing retina exhibited strong deformed accumulations of F-actin at the level of the rhabdomere. In conclusion, overexpression of all human BIN1 isoforms phenocopied dAmphA overexpression during the development of photoreceptor neurons suggesting functional conservation also at the neuronal level.

### Human BIN1iso1 is neurotoxic in Drosophila photoreceptor neurons

**Fig. 2:**
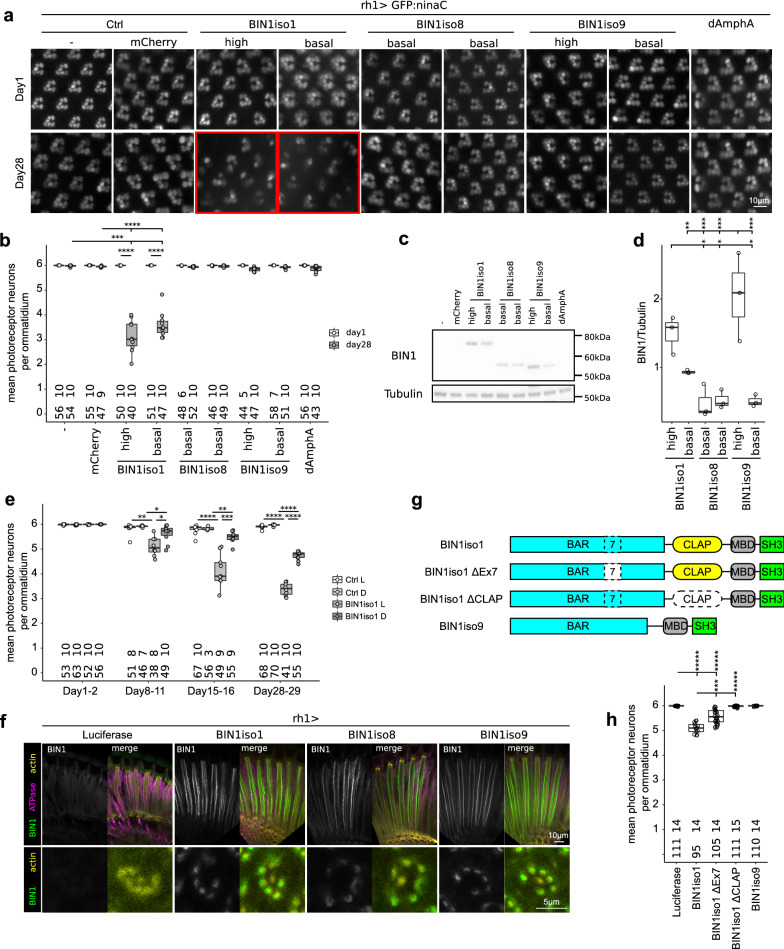
Human BIN1iso1 is neurotoxic in Drosophila photoreceptor neurons. **a** Visualization of retina photoreceptor neurons expressing BIN1 isoforms (rh1 promoter) by cornea neutralization in living flies. **b** Quantification (Kruskal Wallis p = 0.013 followed by Mann–Whitney comparison, ***p < 0.001, ****p < 0.0001). At the bottom of the graph, upper numbers indicate the number of quantified eyes per condition and lower numbers indicate the mean number of ommatidia quantified per eye. Contrary to BIN1iso8 and BIN1iso9, BIN1iso1 expression induced a progressive age-dependent neurodegeneration, which was not dose-dependent. **c**, **d** Western blot analysis of BIN1 isoforms expression in the retina and quantification (n = 3, ANOVA F-value = 12.98, Df = 5, p = 0.00017, with post-hoc Tukey, *p < 0.05, **p < 0.01, ***p < 0.001). **e** Quantification of BIN1iso1-induced photoreceptor neuron degeneration over 4 weeks under a 12 h/12 h light/dark cycle (Ctrl L and BIN1iso1 L) or under constant darkness (Ctrl D and BIN1iso1 D) (Kruskal Wallis p = 0.0005344 for Day8-11, p = 3.264 × 10^−05^ for Day15-16, p = 1.36 × 10^−07^ for Day28-29, followed by Mann Whitney comparison, *p < 0.05, **p < 0.01, ***p < 0.001, ****p < 0.0001). Loss of light did not prevent neurodegeneration although the intensity of degeneration was reduced. **f** Localization of BIN1 isoforms in one-day-old fly photoreceptor neurons. Luciferase was used as a control. Na/K ATPase staining labelled the plasma membrane and actin staining mostly labelled rhabdomere of photoreceptor neurons. Upper panels are longitudinal views of retina, whereas lower panels exhibit sectional views of ommatidium. **g**, **h** Scheme of the truncated BIN1iso1 tested protein and quantification of their toxicity in 15 day-old flies (Kruskal Wallis p = 5.686 × 10^−11^ followed by Mann Whitney comparison, ***p < 0.001, *****p < 0.00001). Loss of the CLAP domain totally abrogated BIN1iso1 toxicity

The neurotoxic effects of human BIN1 isoforms in the developing Drosophila prompted us to investigate whether a similar effect could also be observed after expression of BIN1 isoforms in adult Drosophila. To do so, we employed the outer photoreceptor-specific driver Rh1, active in photoreceptor neurons after rhabdomere morphogenesis at the end of pupal development. We quantified the number of outer photoreceptor neurons following cornea neutralization and rhabdomere visualization. We observed that young flies (1 day-old) had a normal number of outer photoreceptor neurons (6 per ommatidium) with normal morphology (Fig. 2a, b). These observations indicated that the use of rh1 driver bypasses the developmental retinal defects observed using the GMR driver. We then observed that 4-week-old flies expressing BIN1iso1, but not the ones expressing BIN1iso8, BIN1iso9 and dAmphA, lost around half of their outer photoreceptor neurons (Fig. 2a, b). This phenotype was not dose-dependent as flies expressing basal or high BIN1iso1 levels had a similar outer photoreceptor neuron loss. Of note, flies expressing high levels of BIN1iso9 had nearly no loss of outer photoreceptor neurons (Fig. 2a–d). In conclusion, expression of BIN1iso1 induced a progressive neurodegeneration in adult Drosophila photoreceptor neurons and this effect was isoform-specific and not dose-dependent.

Since photoreceptor neurons are specialized neurons highly dependent on the phototransduction cascade [64], we wondered if a defect in this phototransduction cascade may be the cause of the neurodegeneration. For this purpose, we assessed whether the degeneration was light-dependent. BIN1iso1-expressing flies were allowed to age in the normal 12 h/12 h light/dark cycle or under constant dark condition for 4 weeks. Light absence did not prevent outer photoreceptor neurodegeneration even if occurring to a lesser extent (Fig. 2e). This indicated that the phototransduction cascade was not the main cause of the neurodegeneration and that increased global neuronal activity favors BIN1iso1-induced neurodegeneration.

We asked why BIN1iso1 was more toxic for photoreceptor neurons than the other BIN1 isoforms. We wondered whether it could originate from the subcellular localization of BIN1 isoforms. We labelled retinas of 1-day old flies expressing either BIN1iso1, BIN1iso8 or BIN1iso9 for BIN1 (Fig. 2f). All BIN1 isoforms were similarly enriched at the base of photoreceptor neuron rhabdomeres. We next wondered what in the sequence of BIN1iso1 makes it neurotoxic. BIN1iso1 differs from BIN1iso9 by two sequences: (i) the Exon7 in the BAR domain and (ii) the neuronal specific CLAP domain. We tested the neurotoxicity of truncated BIN1iso1 forms for these two sequences (Fig. 2g) after generating the corresponding transgenic flies (Additional file 4: Fig. S3) and observed that loss of the CLAP domain abrogated outer photoreceptor neurodegeneration contrary to the loss of Exon7, which only partially rescued photoreceptor neurons (Fig. 2h). Hence, the BIN1iso1-induced degeneration depends on its CLAP domain and to a lesser extent on the Exon7 in the BAR domain. Since the CLAP domain is known to interact with AP-1 adaptin, AP-2 adaptin and the Clathrin Heavy Chain, which are involved in endocytosis and intracellular trafficking [26, 50, 65], this suggested that the cause of the degeneration could be a defect in endocytosis/intracellular trafficking.

### BIN1iso1 induces vesicle accumulation in photoreceptor neurons

**Fig. 3:**
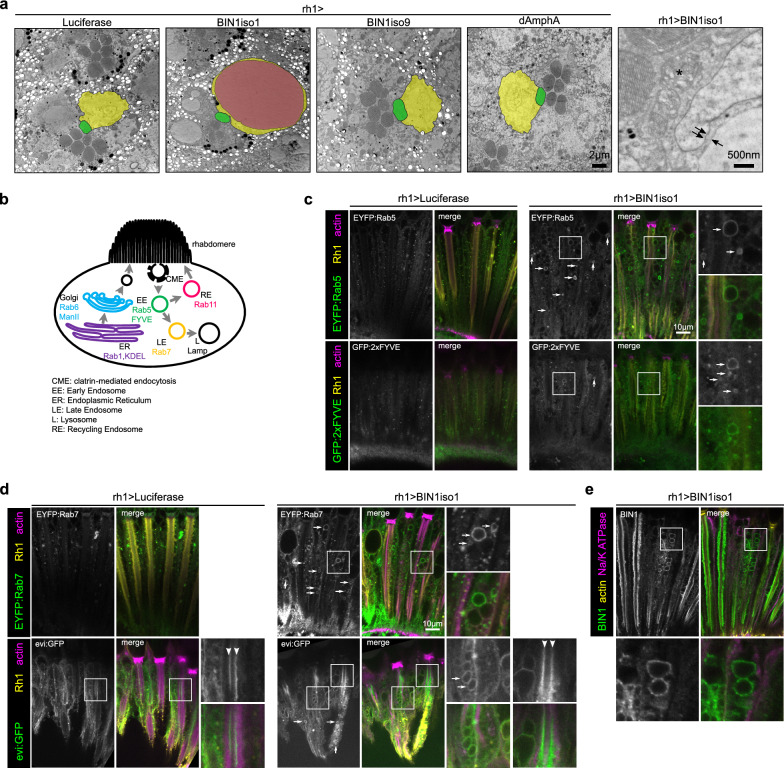
BIN1iso1-induced degeneration is characterized by a strong accumulation of vesicles showing endosomal markers. **a** Electron microscopy images of retina expressing Luciferase (Ctrl), AmphA, BIN1iso1 and BIN1iso9 from 15-day-old flies. 1 to 2 ommatidia transversal sections are seen on the 4 left images. One photoreceptor neuron is outlined per image, its cytoplasm and nucleus being highlighted in yellow and its rhabdomere in green. Each ommatidium contains 6 outer photoreceptor neurons whose rhabdomeres are organized in a trapezoidal shape, with a 7th rhabdomere in the middle corresponding to one of the two inner photoreceptor neurons. In the BIN1iso1 condition, only 4 rhabdomeres can be seen. In addition, the cytoplasm of the photoreceptor is filled with vesicles, some of them (highlighted in magenta) are very big. These vesicles are surrounded by a single membrane (arrow on the right panel) as compared to the nuclear envelope (double arrow). Their rhabdomere is usually disintegrating (*). **b** Scheme of the organelles tested in photoreceptor neurons with the markers used. **c**, **d** Images of photoreceptor neurons expressing BIN1iso1 and EYFP-tagged endogenous Rab5 and GFP:2xFYVE, as early endosome markers (**c**), or EYFP-tagged endogenous Rab7 and evi:GFP, as late endosome/multivesicular body markers (**d**). Rh1 and actin staining, respectively yellow and magenta in the merge image, are used to label retina structure of 15-day-old flies. The photoreceptor neurons expressed Luciferase as a control or BIN1iso1. In the BIN1iso1 conditions, many vesicles were positive for the tested organelle markers (arrows, green staining in merge images). The extracellular inter-rhabdomeric staining for the evi:GFP marker (arrowhead) corresponds to exosomes. **e** Images of photoreceptor neurons expressing BIN1iso1 stained for BIN1, the photoreceptor neuron plasma membrane Na/K ATPase and actin in 7-day-old flies. BIN1iso1 is localized at the base of the rhabdomeres and around some abnormal vesicles as seen in the inset

To further understand the cause of the BIN1iso1-induced degeneration, we analyzed the degenerating photoreceptor neurons by electron microscopy. While neurons in 15-day-old flies either expressing luciferase, BIN1iso9 or dAmphA did not show any abnormalities, the degenerating neurons in BIN1iso1 flies exhibited a strong accumulation of vesicles (Fig. 3a and Additional file 5: Fig. S4a). These vesicles were of various sizes, some of them nearly as big as a complete ommatidium. The cytoplasm of some photoreceptor neurons was filled with vesicles, compacting it against the plasma membrane, which seemed intact. The vesicles were surrounded by a single membrane and not a double membrane as observed in autophagosome (Fig. 3a) and did not have any specific content. Rhabdomeres of BIN1iso1 photoreceptor neurons were disintegrated, whereas the chromatin seemed normal although the nucleus was frequently squeezed in between vesicles (Fig. 3a and Additional file 5: Fig. S4b). Eventually, photoreceptor neurons died (Additional file 5: Fig. S4d). Hence, ultrastructural analysis of degenerating photoreceptor neurons indicated that the neurodegeneration induced by BIN1iso1 is characterized by a strong accumulation of single membrane vesicles of unknown origin.

We next evaluated the nature of these vesicles by immunofluorescence using specific organelle GFP- or YFP-tagged markers for endoplasmic reticulum, Golgi, plasma membrane, early endosome, late endosome/multivesicular body, recycling endosome, lysosome and autophagosome [16] (Fig. 3b and Additional file 6: Fig. S5). In 15-day-old flies, many BIN1iso1-induced vesicles were positive for EYFP:Rab5 and GFP:2xFYVE, markers of early endosome and for EYFP:Rab7 and evi:GFP, markers of late endosome/multivesicular body (Fig. 3c, d). These different markers labelled small- to middle- sized vesicles with the exception of evi:GFP which tended to label bigger vesicles. Some big vesicles were also exceptionally labelled by the lysosomal Lamp2:GFP marker and corresponded to rare multilamellar bodies observed by electron microscopy (Additional file 5: Fig. S4c and Additional file 6: Fig. S5g). Of note, we also noticed that the extracellular space between rhabdomeres of the 8 photoreceptor neurons in the middle of ommatidia, called the interrhabdomeric space, was positive for evi:GFP in both the control and BIN1iso1 conditions (arrowhead Fig. 3d). Since evi:GFP labels exosomes either within the multivesicular bodies or in the extracellular environment [3, 36], evi:GFP interrhabdomeric staining likely corresponded to released exosome which did not appear to be compromised by BIN1iso1 expression. In addition, we observed staining for BIN1 around some vesicles suggesting a potential direct action of BIN1iso1 on the membrane dynamics of these vesicles (Fig. 3e). In conclusion, these results suggested that BIN1iso1-induced vesicles accumulation originated from a blockade at the level of early endosome and/or late endosome.

### BIN1iso1 induces neurodegeneration through blockade of the early endosome trafficking in drosophila photoreceptor neurons

**Fig. 4:**
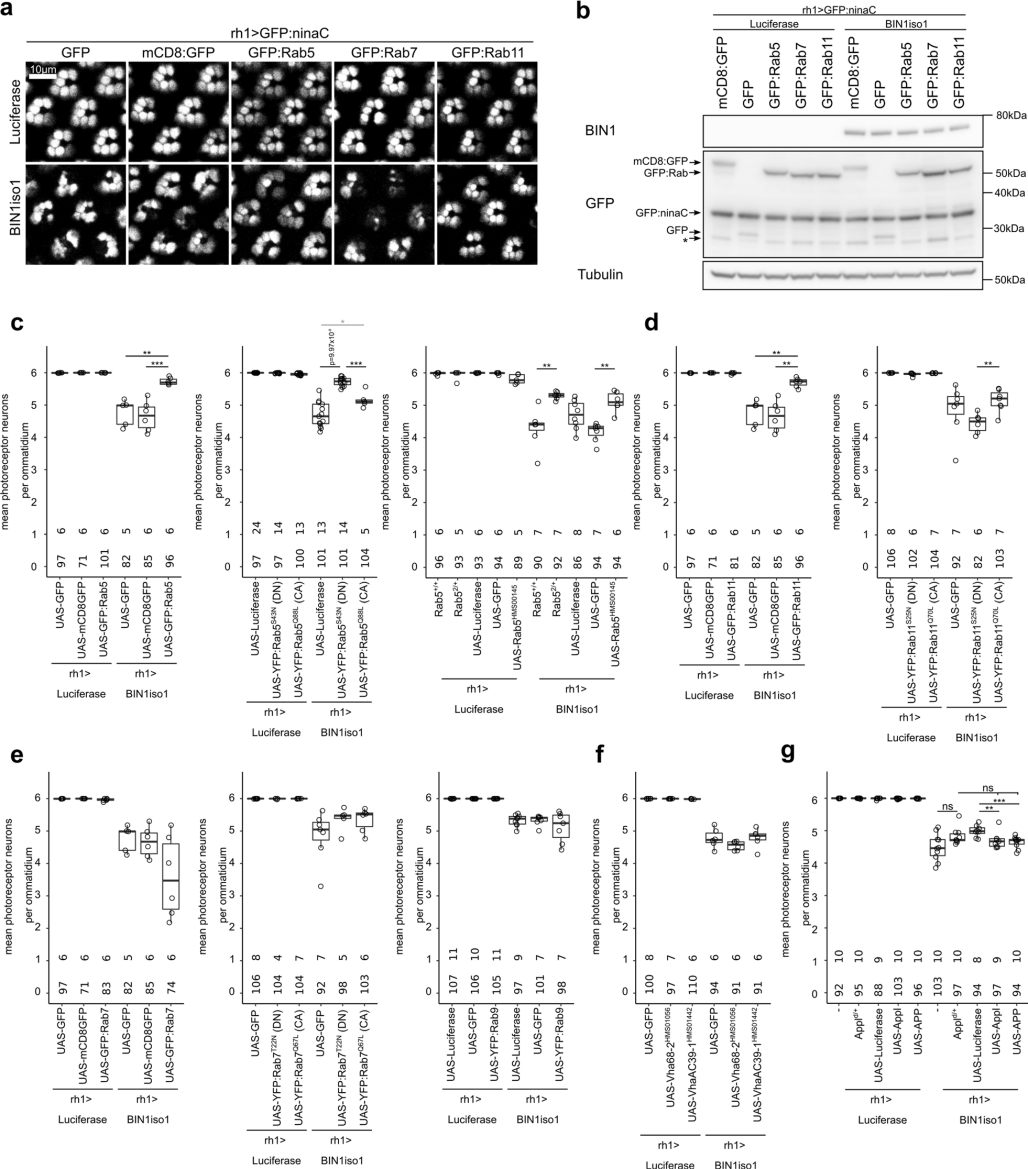
BIN1iso1-induced neurodegeneration is rescued by regulators of the intracellular trafficking. **a** Representative images of 15-day-old retina expressing BIN1iso1 or luciferase (as a control) and constructs modulating Rab5, Rab7 and Rab11 activities. **b** Western blot analysis of BIN1, GFP derivatives in the corresponding conditions showing that BIN1 is not decreased in the conditions in which photoreceptor neurons are rescued. *Non-specific band. **c**–**g** Quantification of the BIN1iso1-induced neurodegeneration upon respective modulation of the early endosome regulator Rab5 activity, the recycling endosome regulator Rab11 activity, the late endosome regulator Rab7 and Rab9 activities, the lysosomal ATPase activity and Appl/APP. At the bottom of the graph, upper numbers indicate the number of quantified eyes per condition and lower numbers indicate the mean number of ommatidia quantified per eye. Statistical analysis was performed using a Kruskal Wallis test (p = 0.003643 for Rab5, p = 6.169 × 10^−06^ for Rab5^DN^ and Rab5^CA^, p = 0.0001721 for Rab5 mutant and knockdown, p = 0.003643 for Rab11, p = 0.03487 for Rab11^DN^ and Rab11^CA^, p = 0.1408 for Rab7, p = 0.24 for Rab7^DN^ and Rab7^CA^, p = 0.8959 for Rab9, p = 0.1394 for lysosomal ATPase subunit knockdown, p = 0.006947 for Appl/APP) followed by Mann Whitney comparison (*p < 0.05, **p < 0.01, ***p < 0.001)

To test if the intracellular trafficking defects were responsible for the neurodegeneration phenotype, we tested if regulators of endosome trafficking could rescue BIN1iso1-induced neurodegeneration. We tested regulator of early endosome (Rab5), recycling endosome (Rab11), late endosome (Rab7 and Rab9) and lysosome (subunits of the V-ATPase) (mostly a collection of UAS transgenes expressing wild-type, constitutively active and dominant negative Rab proteins [68]). We observed an inhibition of BIN1iso1-induced neurotoxicity through a rescue of photoreceptor neurons for regulators of the early endosome Rab5 and recycling endosome Rab11 (Fig. 4a, c, d). Modulation of the late endosome regulators Rab7 and Rab9, and of lysosome V-ATPase did not modify BIN1iso1-induced neuronal loss (Fig. 4e, f). Of note, we checked by western blot that the rescue effect of Rab5 and Rab11 was not due to a decrease in BIN1iso1 expression, consecutive to a dilution of the Gal4 between the multiple UAS constructs (Fig. 4b). We further tested the constitutively active (CA) and dominant negative (DN) forms of Rab5 and Rab11, respectively named Rab5^CA^, Rab5^DN^, Rab11^CA^ and Rab11^DN^. Surprisingly Rab5^DN^ rescued BIN1iso1-expressing photoreceptor neurons, whereas Rab5^CA^ had no effect (Fig. 4c). This indicated that, although counterintuitive, overexpression of wild-type Rab5 resulted in a loss of function of Rab5 and that loss of function of Rab5 is protective against BIN1iso1-induced neurodegeneration. We confirmed this result when one copy of Rab5 (Rab5^2/+^) was removed or by knocking down Rab5 (Rab5^HMS00145^) (Fig. 4c). Contrary to Rab5, Rab11^DN^ increased BIN1iso1-induced neuronal loss (although not significant) (Fig. 4d). A gain of function of Rab11 seemed, therefore, protective against BIN1iso1-induced neurodegeneration. We also tested the fast recycling endosome regulator Rab4 (Additional file 7: Fig. S6). Although one construct expressing mRFP-tagged Rab4 rescued BIN1-induced neurodegeneration, another YFP-tagged Rab4 construct did not, and neither did the YFP-tagged Rab4^DN^ or Rab4^CA^ construct. Altogether, because the neurodegeneration was rescued by modulation of regulators of early and recycling endosomes, these results indicated that BIN1iso1-induced photoreceptor neuron degeneration is due to a defect in the early endosome trafficking.

We further wondered if BIN1iso1 neurotoxicity may be mediated by APP or one of its metabolites. Indeed, APP-β Carboxy Terminal Fragment (APP-βCTF), the product of APP cleavage by β-secretase and precursor of Aβ peptide through γ-secretase cleavage, induces endosome enlargement in neurons [28]. Drosophila has a single homolog of APP, called APP Like (Appl), which is also processed by several secretases and generates secreted fragments, a neurotoxic Aβ-like peptide and an C-terminal intracellular domain (AICD) [7]. Interestingly, Appl regulates endolysosomal function in Drosophila neurons [30]. In addition, some results support a regulation of APP processing by BIN1 [62]. We tested if loss of function, overexpression of *Appl* or overexpression of human APP could modulate BIN1iso1-induced photoreceptor neuron degeneration. Loss of one copy of *Appl* had no effect suggesting that *Appl* is not required for BIN1iso1 toxicity (Fig. 4g). Overexpression of *Appl* and human *APP* significantly enhanced PR loss but only slightly in terms of fold change and not significantly compared to the loss of one copy of *Appl* (Fig. 4g). Overall, these experiments suggest that APP (and potentially its metabolites) do not contribute to the BIN1iso1 neurotoxicity observed in our Drosophila model.

### Generation and characterization of *BIN1* WT and KO human induced neurons

**Fig. 5:**
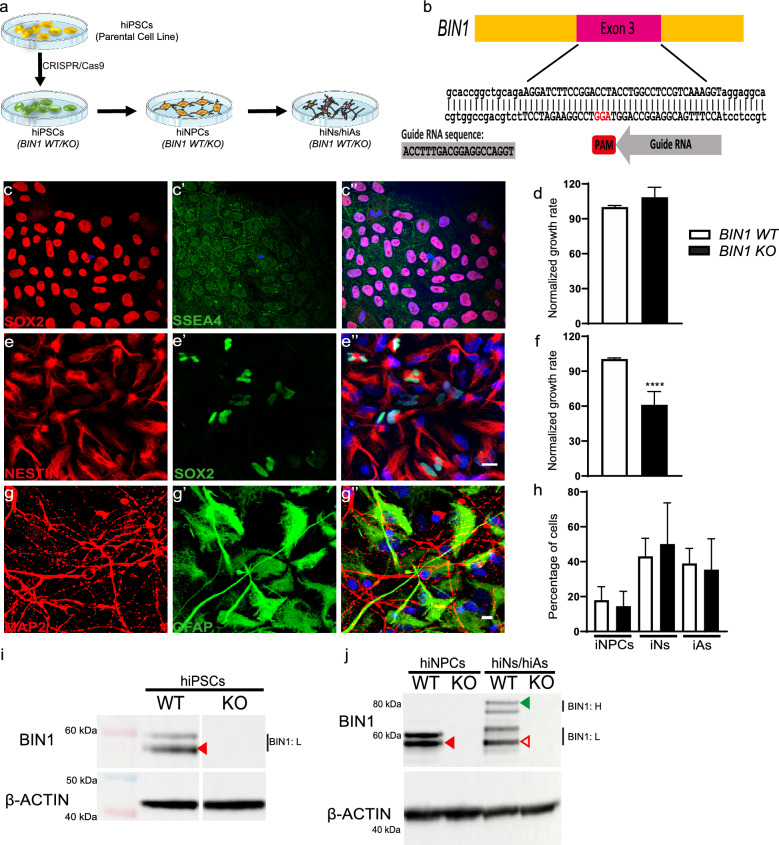
Characterization of hiPSCs and their cell derivatives. **a** Schematic showing the production of *BIN1* WT and KO hiPSCs from parental cell line using CRISPR/Cas9 technology. These hiPSCs, in turn, were used to generate intermediate hiNPCs, and subsequently, mixed cultures of hiNs and hiAs. **b** Representation of exon 3 region of BIN1 was targeted for the production of *BIN1* WT and KO hiPSCs by CRISPR/Cas9 technology. The guide RNA sequence is shown (grey). **c**-**c**’’ Representative images showing pluripotency markers SOX2 (red), SSEA4 (green) and stained with Hoechst 33258 (**c**’’) in *BIN1* WT and KO hiPSCs. **d** Plot showing the normalized growth rate of *BIN1* WT and KO hiPSCs (N = 4 independent cell passages; p = 0.77, Unpaired t-test). **e**-**e’’** Representative images showing hiNPCs labelled for NESTIN (red), SOX2 (green) and stained with Hoechst 33258 (e’’, Scale Bar = 20 µm). **f** Plot showing the normalized growth rate of *BIN1* WT and KO hiNPCs (N = 9 independent cell passages: ****p < 0.0001, Unpaired t-test). **g**-**g’’** Representative images showing a 6-week-old mixed hiNs/hiAs culture labelled for the neuronal marker MAP2 (red), the astrocytic marker GFAP (green) and stained with Hoescht 33258 (g’’, Scale Bar = 10 µm) **h** Plot showing the percentage of cells in different cell populations – hiNPCs, hiNs, and hiAs (ANOVA _F(5,12)_, p = 0.45). **i** Immunoblot for BIN1 and actin in BIN1 WT and KO hiPSCs samples. Band indicated on the blot for the WT cells (solid red arrowhead) indicates BIN1-light (BIN1:L) isoforms of BIN1. **j** Immunoblot showing the expression of BIN1:L isoforms (solid red arrowhead) in WT hiNPCs and both BIN1:L (hollow red arrowhead) and BIN1-heavy (BIN1:H; solid green arowhead) isoforms in 6-week-old mixed cultures of hiNs and hiAs

Next, we wondered whether the role of BIN1iso1 in endosome trafficking was conserved in Human. To address this possibility, we generated human isogenic *BIN1* wild type (WT) and knockout (KO) pluripotent stem cell (hiPSC) lines by CRISPR/Cas9 technology. Both hiPSC lines showed similar expression of pluripotency cell markers (SOX2 and SSEA4, Fig. 5c) and growth rates (Fig. 5d). *BIN1* WT hiPSC expressed low molecular weight BIN1 isoforms, including likely BIN1iso9 (Fig. 5i). We then employed these cells to generate hiPSC-derived neurons both in bi-dimensional (2D) cell cultures and in cerebral organoids.

Following neural induction, both *BIN1* WT and KO human induced neural progenitor cells (hiNPCs) expressed similar levels of NESTIN and SOX2 (Fig. 5e). Although *BIN1* KO hiNPCs showed a significant reduction in growth rate when compared to WT (Fig. 5f), both hiNPCs lines were expanded up to 10 passages and readily generated similar proportions of human induced neurons (hiNs) and astrocytes (hiAs) when subjected to conditions of differentiation (Fig. 5g, h). WT hiNPCs mainly expressed low molecular weight BIN1 isoforms, whereas in differentiated cultures both low and high molecular weight isoforms, probably corresponding to BIN1iso9 and BIN1iso1 respectively, were expressed (Fig. 5j). The expression pattern observed for BIN1 isoforms likely reflects the mixed composition of the differentiated cell cultures at 6 weeks, comprising both neurons and astrocytes, which mainly express BIN1iso1 and BIN1iso9, respectively [69].

**Fig. 6:**
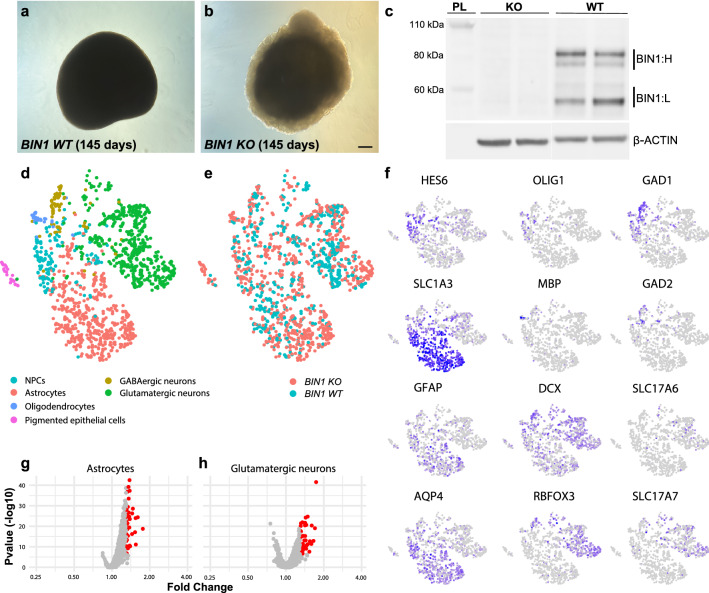
*BIN1* WT and KO cerebral organoids show similar composition and gene expression. **a**, **b** Brightfield images of a *BIN1* WT (a) and KO (b) cerebral organoids after 145 days (d) in culture. Calibration bar: 200 µm. **c** Western blot for BIN1 and ACTIN in 190 days-old *BIN1* WT and KO cerebral organoids used for snRNA-seq. Bands corresponding to light (BIN1:L) and heavy BIN1 (BIN1:H) isoforms are indicated. MW – molecular weight. **d**, **e** tSNE plots of all 2990 cells from the snRNA-seq color-coded by cell type annotation (d) and genotype (e). **f** tSNE plots showing the expression of neural progenitor cell (HES6 and SLC1A3), astrocytes (SLC1A3, GFAP and AQP4), oligodendrocytes (OLIG1, MBP), pan neuronal (DCX, RBFOX3), GABAergic neurons (GAD1 and GAD2) and glutamatergic neurons (SLC17A6 and SLC17A7) markers. **g**, **h** Volcano plots showing genes differentially expressed in *BIN1* KO astrocytes (g) and glutamatergic neurons (h) compared to WT cells. Red dots indicate genes with fold change (FC) > 1.3 and false discovery rates (FDR) < 0.01

Likewise, *BIN1* WT and KO hiPSCs were able to generate cerebral organoids with no obvious differences in size and composition (Fig. 6a, b). Expression of BIN1 protein in 190-day-old *BIN1* WT cerebral organoids was confirmed by western blot and showed a similar pattern as the one described in 2D cell cultures (Fig. 6c). Next, using single-nucleus RNA-sequencing (snRNA-seq), we observed that cerebral organoids of both genotypes contain all major neural cell types, with no significant difference in the proportions of cell types (Fig. 6d, e, f and Additional file 8: Fig. S7). We then performed differential gene expression analysis for all different cell types identified in *BIN1* WT and KO cerebral organoids using DESeq2 [38]. We observed 41 differentially expressed genes (DEGs; 0.7 < FC > 1.3 and FDR < 0.01) in glutamatergic neurons and 43 DEGs in astrocytes (Fig. 6g, h and Additional file 9: Table S1). All the other cell types showed none or only 1–2 DEGs (Additional file 9: Table S1), indicating that *BIN1* WT and KO neural cells have similar gene expression profiles. Importantly, even for the 41 DEGs observed in glutamatergic neurons, no significant enrichment for gene ontologies associated with endocytic pathway was observed (Additional file 10: Table S2). These findings indicate that *BIN1* WT and KO hiPSC-derived neurons mainly differ by the expression of BIN1. As a consequence, potential defects in the endosome pathway in these cell models are likely not due to major transcriptional modifications but due to a direct action of BIN1 protein and its interaction with other proteins.

APP metabolism and endosome trafficking are tightly interconnected processes [23, 34] and previous work has suggested a role for BIN1 in their regulation [42, 62]. To probe whether BIN1 deletion could affect amyloidogenic APP processing in hiNs, we measured the levels of the APP β- CTF in cerebral organoids and 2D cell cultures (Additional file 11: Fig. S8). We found a significant reduction in the levels of APP β-CTF normalized either by ACTIN or full-length APP in BIN1 KO compared to WT cerebral organoids (Additional file 11: Fig. S8a, b). A similar trend was observed in 2D cell cultures, but without reaching statistical significance (Additional file 11: Fig. S8c, d).

### *BIN1* null mutation is associated with smaller early-endosome vesicles in hiPSC-derived neurons

**Fig. 7:**
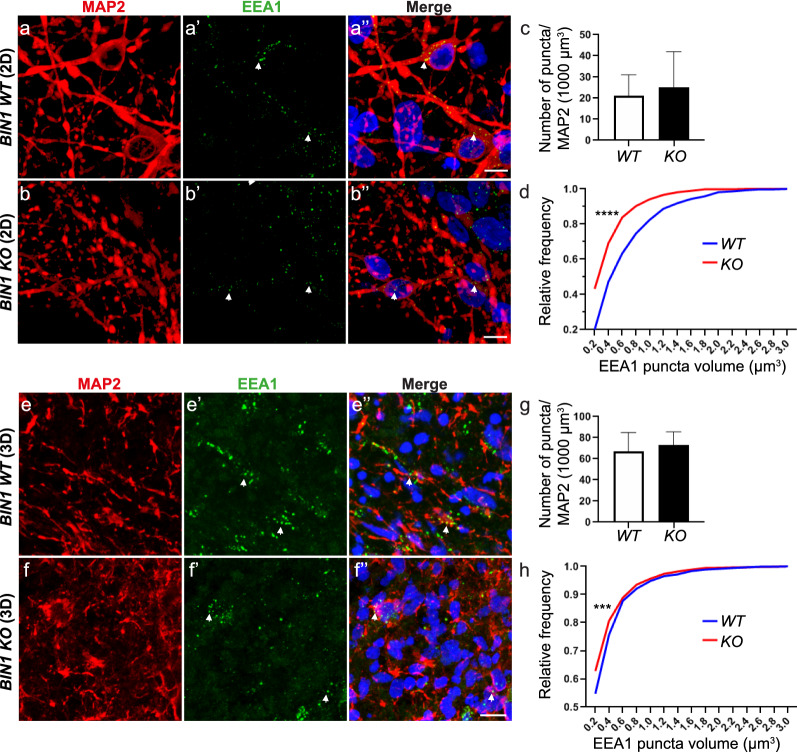
Increased proportion of small-volume endosomes in *BIN1* null mutant hiNs. **a**– **b’’** Representative images of *BIN1* WT and KO hiNs in 6-week-old 2D cultures immunolabeled with antibodies against MAP2 (red, a and b) and EEA1 (green, a’ and b’) and stained with Hoechst 33258 (blue, a’’ and b’’) (Scale Bar = 10 µm). **c** Plot showing the quantification of the number of EEA1^+^ puncta per 1000 µm^3^ of MAP2^+^ surface (N = 3 independent cell cultures; *BIN1* WT hiNs: 21.15 ± 9.94 EEA1^+^ puncta per 1000µm^3^ of MAP2^+^ surface; *BIN1* KO hiNs: 25.31 ± 16.58 EEA1^+^ puncta per 1000µm^3^ of MAP2^+^ surface; p = 0.84, Unpaired t-test). **d** Plot showing the cumulative distribution of EEA1^+^ puncta volumes in *BIN1* WT (blue line) and KO (red line) hiNs (N = 3 independent cell cultures; Kolmogorov–Smirnov test, ****p < 0.0001). **e**–**f’’** Coronal sections of 190-days-old *BIN1* WT and KO cerebral organoids immunolabeled with antibodies against MAP2 (red, e and f) and EEA1 (green, e’ and f’) and stained with Hoechst 33258 (blue, e’’ and f’’). **g** Quantification of the number of EEA1^+^ puncta per 1000 µm^3^ of MAP2^+^ surface (N = 3 organoids per genotype; cerebral organoids: *BIN1* WT hiNs: 66.72 ± 18.13 EEA1^+^ puncta per 1000µm^3^ of MAP2^+^ surface; *BIN1* KO hiNs: 73.18 ± 12.47 EEA1^+^ puncta per 1000µm^3^ of MAP2^+^ surface; Unpaired t-test, p = 0.96). **h** Cumulative distribution of EEA1^+^ puncta volumes in *BIN1* WT (blue line) and KO (red line) hiNs (N = 3 organoids per genotype; Kolmogorov–Smirnov test, ***p < 0.001)

In order to probe the impact of *BIN1* null mutation in hiNs, we quantified the number and size of the early endosome antigen 1 (EEA1)-expressing endosomes in microtubule-associated protein 2 (MAP2)-expressing cells in both 2D cultures and cerebral organoids (Fig. 7). EEA1 is an early endosomal Rab5 effector protein that has been implicated in the docking of incoming endocytic vesicles before fusion with early endosomes [43]. We observed a significant change in the cumulative distribution of EEA1^+^ endosome volumes in hiNs both in 2D cultures and cerebral organoids, mostly due to a predominance of small volume endosomes in *BIN1* KO compared to WT hiNs (Fig. 7d, h). Conversely, no significant change in the number of endosomes was observed in hiNs of both genotypes (Fig. 7c, g). These data suggested that BIN1 is involved in the regulation of early endosome size in human neurons.

### BIN1iso1 specifically modulates sizes of the early-endosome vesicles

**Fig. 8:**
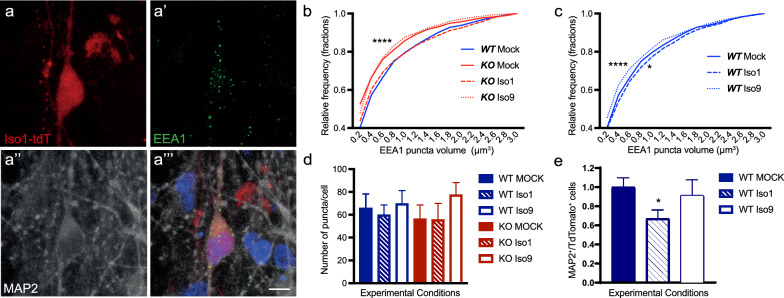
Rescue of endosomal phenotype in *BIN1* null mutant neurons by transduction of BIN1 Isoform 1. **a**–**a’’’** Representative images of *BIN1* WT and KO hiNs in 6-weeks-old 2D culture immunolabeled with antibodies against tdTomato (tdT, red, a), EEA1 (green, a’), MAP2 (grey, a’’), and stained with Hoechst 33258 (blue, a’’’) (Scale Bar = 10 µm). **b** Plot showing the cumulative distribution of EEA1^+^ puncta volumes in transduced *BIN1* KO hiNs with tdT lentiviral constructs; *BIN1iso1* (dashed red line), *BIN1iso9* (dashed red line), and Mock-tdT (solid red line). *BIN1* WT hiNs tranduced with Mock-tdT (solid blue line) is indicated to show the rescue effect of the BIN1iso1 construct (orange line) in *BIN1*-null mutant neurons. Kolmogorov–Smirnov test followed by Bonferroni correction: *BIN1* WT + Mock vs *BIN1* KO + Mock: ****Padj < 0.0001; *BIN1* WT + Mock vs *BIN1* KO + iso9: Padj < 0.0001; *BIN1* KO + Mock vs *BIN1* KO + Iso1: ****Padj < 0.0001; *BIN1* WT + Mock vs *BIN1* KO + Iso1: Padj = 0.174; *BIN1* KO + Mock vs *BIN1* KO + Iso9: Padj = 0.207 (N = 3 independent cell cultures). **c** Plot showing the cumulative distribution of EEA1^+^ puncta volumes in transduced *BIN1* WT hiNs with tdTomato (tdT)-expressing lentiviral constructs; *BIN1iso1* (dashed blue line), *BIN1iso9* (dotted blue line), and Mock-tdT (solid blue line). Kolmogorov–Smirnov test followed by Bonferroni correction: *BIN1* WT + Mock vs *BIN1* WT + Iso1: *Padj = 0.04; *BIN1* WT + Mock vs *BIN1* WT + Iso9: ****Padj < 0.0001 (N = 3 independent cell cultures). **d** Graph showing the quantification of numbers of EEA1 puncta per cell in cells transduced with tdT-tagged lentiviral constructs (N = 3 independent cell cultures. EEA1 ^+^ puncta/neuron: *BIN1* WT + Mock: 65.86 ± 12.78; *BIN1* WT + Iso1: 59.75 ± 8.895; *BIN1* WT + Iso9: 69.82 ± 11.42; *BIN1* KO + Mock: 56.47 ± 12.27; *BIN1* KO + Iso1: 55.65 ± 14.52; *BIN1* KO + Iso9: 77.31 ± 11.04; p = 0.81, ANOVA _F(5,90)_). **e** Quantification of MAP2^+^ /tdTomato^+^ neurons in BIN1iso1- and BIN1iso9-transduced cells relative to Mock-tdT-transduced cells (N = 3 independent cell cultures; Number of tdT^+^ cells: Mock = 468; BIN1iso1 = 193; BIN1iso9 = 103; ANOVA followed by Dunnett’s multiple comparisons test, **p = 0.0289)

We then wondered whether the function of BIN1 in endocytosis could also be isoform-specific in human neurons, as observed in Drosophila. To test this possibility, we set out to perform lentiviral-mediated transfection with fluorophore-expressing BIN1iso1, BIN1iso9 or control plasmids in hiNs at 3 weeks of differentiation. After 3 additional weeks, we quantified the number and size of EEA1-expressing endosomes in transfected hiNs identified by tdTomato expression (Fig. 8). We observed that expression of BIN1iso1, but not BIN1iso9, in *BIN1* KO hiNs fully rescued the volume of EEA1^+^ endosomes to values similar of those observed in WT hiNs (Fig. 8b). We also over-expressed BIN1iso1 and BIN1iso9 in WT hiNs. Consistent with our previous observation in flies, we observed that BIN1iso1 overexpression in neurons led to an increase of large volumes EEA1^+^ endosomes, whereas the opposite effect was observed in neurons overexpressing BIN1iso9 (Fig. 8c). Neither in *BIN1* WT nor in KO hiNs did we observe significant changes in the number of puncta per cell after BIN1iso1 or BINiso9 overexpression (Fig. 8d). Lastly, we evaluated whether BIN1iso1 overexpression could have a neurotoxic effect in hiNs. To that end, we quantified the proportion of MAP2^+^ neurons in 6 weeks cultures after transduction with BIN1iso1-, BIN1iso9- or control-tdTomato^+^ cells. We found that BIN1iso1 overexpression led to a 30% reduction in the proportion of neurons compared to controls (Fig. 8e). Interestingly, this effect of BIN1iso1 was not observed in *BIN1* KO cells, suggesting that only supra-physiological expression levels of this isoform could be toxic for neurons.

Altogether, these observations indicate that BIN1iso1 expression is sufficient to regulate endosome volumes, even in the absence of other BIN1 isoforms in *BIN1* KO hiNs, and that increased expression of BIN1iso1 deregulates endosome size and can lead to degeneration of human neurons, as observed in Drosophila photoreceptors.

## Discussion

In this work, we assessed the function and potential neurotoxicity of human BIN1 isoforms in Drosophila and human neurons. We show a functional conservation of human BIN1 in Drosophila as BIN1iso1, BIN1iso8 and BIN1iso9 phenocopied dAmphA-induced photoreceptor neuron developmental defects and BIN1iso8 was able to rescue the locomotor defects associated with the loss of *dAmph* in Drosophila. Expression of BIN1iso1 resulted in a progressive neurodegeneration of photoreceptor neurons that was isoform-specific and dose-independent. The degeneration did not depend on the activation of the phototransduction cascade and was associated with a strong accumulation of vesicles harboring early and late endosomal markers. These results suggested that a BIN1 isoform-specific alteration of the endosome-lysosome pathway could contribute to neuronal degeneration. Accordingly, the degeneration was prevented by a loss of function of the early endosome regulator Rab5 and a gain of function of the recycling endosomal protein Rab11. Lastly, we observed a conservation of BIN1iso1 function in hiNs in 2D cultures and in organoids. Loss of BIN1 resulted in reduced size of early endosomes, whereas expression of BIN1iso1, but not BIN1iso9, was able to rescue the reduced size of early endosomes in the *BIN1* KO hiNs. As in Drosophila, overexpression of BIN1iso1 resulted in enlarged early endosomes and had neurotoxic effects in WT hiNs.

Our results indicate context- and isoform-specific functions of BIN1: (i) Expression of BIN1iso1, BIN1iso8 and BIN1iso9 altered photoreceptor neuron rhabdomere morphogenesis during development in an isoform-independent and dose-dependent manner, whereas in the adult photoreceptor neurons BIN1iso1 expression induced neurodegeneration in an isoform-dependent and dose-independent manner; (ii) in human neurons, we observed a shift in BIN1 isoform expression from low molecular weight isoforms in hiPSCs and NPCs (likely mainly BIN1iso9) to high molecular weight isoforms in hiNs (likely mainly BIN1iso1); (iii) BIN1iso1 expression rescued the size of BIN1-deficient early endosomes contrary to BIN1iso9. Collectively, these results argue for an important role of BIN1iso1 in mature neuronal cells and support the fact that BIN1iso1 is a neuron-specific isoform with specific functions in this cell-type [52, 69]. Furthermore, (i) *BIN1* KO hiNs showed early endosomes of reduced size; (ii) in *BIN1* KO hiNs, BIN1iso1 expression rescued early endosome sizes but did not lead to enlargement of these vesicles; and (iii) in WT hiNs, BIN1iso1 overexpression induced early endosome enlargement. These results suggest that homeostatic BIN1iso1 expression levels need to be tightly regulated to allow proper endocytic trafficking in neurons. Of note, BIN1iso9 overexpression in WT hiNs induced a reduction of early endosome size. Since BIN1 forms dimers through its BAR domain [51], we postulate that BIN1iso9 may dimerize with BIN1iso1 and inhibit BIN1iso1 function in a dominant negative manner. Interestingly, this may also indicate another level of complexity to regulate BIN1 functions at the protein level.

Our results support a direct role of BIN1iso1 in neurons at the early endosome crossroad as indicated (i) by the labeling of large vesicles with early endosome markers in Drosophila, (ii) by the Rab5 rescue experiment in Drosophila and (iii) by the early endosome size modulation in hiNs. Of note, the phenotype with very large vesicles in Drosophila likely results from Drosophila being a heterologous system for human BIN1iso1, and as a consequence, exhibiting stronger phenotypes. Additionally, in Drosophila, some of the vesicles also exhibited markers for late endosome/MVB and exceptionally, lysosome. However, regulation of late endosome/MVB or lysosomal function did not modulate photoreceptor degeneration and secretion of exosomes did not appear to be blocked, suggesting that BIN1iso1 defect do not occur at this level. BIN1 has been proposed to regulate endocytosis by interaction with clathrin, Adaptor Proteins (AP) and dynamins [12, 22, 26, 50, 65]. Since some of these proteins are also involved in vesicle budding of the intracellular organelles [26], we propose that BIN1iso1 may also inhibit vesicle budding of early endosomes, thus, leading to an increase in their size. Overexpressing Rab11 would overcome this inhibition through the activation of endosome recycling, decompress enlarged endosomes and explain the rescuing effect of a gain of Rab11 that we observed. In support of this direct action of BIN1iso1 on endosomes, (i) we observed BIN1iso1 on the large endosomal vesicles in Drosophila, (ii) BIN1 knockout in cerebral organoids reduced endosome size without impacting expression of genes involved in the endosome trafficking pathway, and (iii) in Drosophila, BIN1iso1 toxicity was dependent on the CLAP domain, which is known to directly bind intracellular trafficking proteins like clathrin and APs [50]. This regulation of early endosomes correlates with a broader role of BIN1 in the regulation of intracellular trafficking ranging from endocytosis [6, 65] to recycling endosomes [62] as well as related processes in neuronal pre- or post-synaptic compartments [53, 56].

Interestingly, a growing body of evidence supports a dysregulation of the endosomal-lysosomal system as a plausible underlying mechanism in AD pathogenesis [45, 58]. Several AD susceptibility genes identified by GWAS (*BIN1, PICALM, EPHA1, CD2AP, SORL1* and *RIN3*) encode proteins that function predominantly in endocytic trafficking [15]. Endosome enlargement has been described to be the first cytopathological marker of AD, before the emergence of plaques and tangles [8]. Several human AD cell models, namely hiNs originating from sporadic and familial AD patients [27], hiNs carrying fAD APP and PSEN1 mutations [34] and hiNs knocked-out for the AD risk gene SORL1 [25, 32], recapitulate these endosomal defects similarly to what we observed in the BIN1iso1 overexpressing hiNs. In AD and Down syndrome, these defects are associated with an overactivation of Rab5 [47] and overactivation of Rab5 in mice mimics AD-like endosomal dysfunction [48]. We have also shown that BIN1iso1 effects in Drosophila are Rab5-dependent. Early endosomes are a major site of APP processing by β-secretase to yield APP β-CTF. Therefore, alterations in endocytic pathways can affect APP metabolism and this likely explain the decrease in APP β-CTF we observed in the BIN1 knocked-out hiNs. Reciprocally, APP β-CTF mediates endosomal defects in fAD mutant hiNs [34]. To do so, APP β-CTF activates Rab5 via APPL1 [31]. However, in our study, loss of *Appl*, the Drosophila ortholog of APP, did not rescue BIN1iso1-induced neurodegeneration, suggesting that APP β-CTF does not mediate BIN1iso1 toxicity. The involvement of APP β-CTF have also been questioned in SORL1 knocked-out hiNs with contradictory results in two studies showing APP β-CTF-dependent or -independent endosomal defects [25, 32]. Another putative intermediate is Ras and Rab Interactor 3 (RIN3), a guanine nucleotide exchange factor (GEF) for the Rab5 small GTPase family, which is located in a GWAS-defined AD susceptibility locus [35]. Upregulation of RIN3 induces endosomal dysfunction through Rab5 [57] and RIN3 interacts with BIN1 [29, 57]. Further experiments would be, thus, of interest to test RIN3 as an intermediate for BIN1iso1 neurotoxicity. Finally, we show that BIN1iso1 endosomal defects and toxicity can be attenuated by a gain of function of Rab11, which dovetails with data showing a colocalization of BIN1 with Rab11 [56] and indicating that Rab11 activity is altered in AD in relationship with endosomal trafficking [5, 63, 66]. Overall, our results strongly support a role of BIN1 in the endosomal dysregulation observed in AD.

Since we observed endosome enlargement and neurodegeneration upon BIN1iso1 overexpression, our results also suggest that an increase in BIN1iso1 may be deleterious to neurons and contribute to early phases of AD pathology through early endosome alterations. It implies an increase in BIN1iso1 levels in neurons, which remains elusive. To date, most experiments showing altered BIN1 expression in the AD brain have focused in samples obtained at late stages of the pathology [1, 9, 21, 24, 41, 52, 60] and none have directly measured the levels of BIN1 isoforms at early stages of the pathology at the single-cell resolution. Therefore, at this point, it is not possible to establish a clear link between levels of BIN1 expression in neurons and AD pathogenesis. Due to the numerous biological roles of BIN1 in AD pathophysiology, it is parsimonious to envisage that both up- and down-regulation of BIN1 isoforms expression in the human brain could affect disease progression through different cell-type specific mechanisms, including the endosomal abnormalities described here.

Future studies should also address the possibility that *BIN1* polymorphisms associated with an increased AD risk could affect the expression of BIN1iso1 in neurons. Interestingly, the variant rs59335482, an insertion allele associated with a higher AD risk, is able to increase transcriptional activity in a luciferase assay in vitro using HEK cells and SH-SY5Y neuroblastoma cells, and is also associated with an increase in *BIN1* mRNA expression in the brain [9]. However, individual BIN1 isoforms were not analyzed in this study. Other variants, rs6733839 and rs13025717, in linkage disequilibrium with the above-mentioned variant, have been shown to be located in a region enriched in microglia-specific enhancers [10, 46]. Deletion of a large 363 bp promoter region containing these variants in hiPSCs resulted in a decrease of *BIN1* expression specifically in human induced microglia, but not in Neurog2-induced hiNs. Unfortunately, neither the transcriptional effect of the exact variants nor the expression of BIN1 in spontaneously differentiated hiNs have been tested yet. In addition, the possibility that the impact of functional variants may depend on specific AD pathophysiological process, e.g. Aβ exposure, has not been assessed. There is, thus, a high uncertainty about functional variants in the BIN1 locus. Even if our current study was not intended to directly assess functional consequences of AD risk-related BIN1 polymorphisms but to focus on BIN1 isoforms function in neurons, which are still insufficiently described and understood, it may provide a context for BIN1-associated risk, namely the dysregulation of early endosome size and function. Up to now and not exclusive from each other, BIN1 polymorphisms have been associated with Tau but not amyloid loads in post-mortem AD brain tissue [9] and they are consistently associated with faster Aβ-associated Tau-PET accumulation and cognitive decline in AD patient [18, 19].

## Conclusions

In conclusion, an increase of BIN1iso1 in neurons could contribute to AD pathogenesis by increasing the size of early endosomes observed early in the pathogenic process and by inducing neurodegeneration. Other AD genetic determinants have also been shown to regulate early endosome size, supporting early endosome defects as a major event in the pathogenesis of AD.

## Supplementary Information

**Additional file 1**. Supplementary information and supplementary methods.

**Additional file 2**. Figure S1. Creation of transgenic lines expressing human BIN1 isoforms.

**Additional file 3**. Figure S2. Scheme of AmphMI08903-TG4.0 allele.

**Additional file 4**. Figure S3. Generation and test of transgenic Drosophila expressing truncated human BIN1-1 forms for the Exon7 (BIN1-1 ΔEx7) and the CLAP domain (BIN1-1 ΔCLAP).

**Additional file 5**. Figure S4. Electron microscopy images of BIN1-1-induced photoreceptor degeneration in Drosophila eyes.

**Additional file 6**. Figure S5. Screening of organelle markers in BIN1-1-induced degenerating photoreceptor neurons.

**Additional file 7**. Figure S6. Effect of Rab4 modulation on BIN1iso1 neurotoxicity in Drosophila photoreceptor neurons.

**Additional file 8**. Figure S7. Graphic representation of proportions of cell types (in percentages) in *BIN1* KO and WT cerebral organoids.

**Additional file 9**. Table S1. Differential gene expression analysis for all different cell types identified in *BIN1* WT and KO cerebral organoids.

**Additional file 10**. Table S2. Gene ontology enrichment analysis for the 41 DEGs observed in glutamatergic neurons.

**Additional file 11**. Figure S8. Decreased APP/β-CTF levels in BIN1 KO cerebral organoids.

## Data Availability

The datasets analyzed during the current study are available from corresponding authors on reasonable request.

## Abbreviations

Aβ: Amyloid β peptide
AD: Alzheimer’s disease
APP β-CTF: APP β-carboxy terminal fragment
BAR: BIN1/Amphiphysin/RVS
BIN1iso1: Human BIN1 isoform1
BIN1iso8: Human BIN1 isoform8
BIN1iso9: Human BIN1 isoform9
CA: Constitutively active
CLAP: Clathrin and adaptor protein-2 binding
DEG: Differentially expressed genes
DN: Dominant negative
hiA: Human induced astrocyte
hiN: Human induced neuron
hiNPC: Human induced neural progenitor cells
hiPSC: Human induced pluripotent stem cells
KO: Knockout
snRNA-seq: Single-nucleus RNA-sequencing
WT: Wild type
