# Supplementary material for: The Alzheimer susceptibility gene BIN1 induces isoform-dependent neurotoxicity through early endosome defects
Source: Acta Neuropathol Commun. 2022 Jan 8;10:4. doi: 10.1186/s40478-021-01285-5 (PMC8742943; doi:10.1186/s40478-021-01285-5)
Supplement: Supplementary file 1 — Additional file 1. Supplementary information and supplementary methods. [file 40478_2021_1285_MOESM1_ESM.docx]

# Supplementary information to

**The Alzheimer susceptibility gene *BIN1* induces isoform-dependent neurotoxicity through early endosome defects**

Acta Neuropathologica Communications

Erwan Lambert^1,#^, Orthis Saha^1,#^, Bruna Soares Landeira^1^, Ana Raquel Melo de Farias^1,2^, Xavier Hermant^1^, Arnaud Carrier^3^, Alexandre Pelletier^3^, Lindsay Davoine^1^, Cloé Dupont^1^, Philippe Amouyel^1^, Amélie Bonnefond^3^, Frank Lafont^4^, Farida Abdelfettah^1^, Patrik Verstreken^5,6^, Julien Chapuis^1^, Nicolas Barois^4,7^, Fabien Delahaye^3^, Bart Dermaut^8^, Jean-Charles Lambert^1^, Marcos R. Costa^1,2,##,^*, Pierre Dourlen^1,##,^*

1. Univ. Lille, Inserm, CHU Lille, Institut Pasteur de Lille, U1167-RID-AGE facteurs de risque et déterminants moléculaires des maladies liées au vieillissement, DISTALZ, Lille, France
2. Brain Institute, Federal University of Rio Grande do Norte, Natal, Brazil
3. Univ. Lille, Inserm, CNRS, CHU Lille, Institut Pasteur de Lille, U1283-UMR 8199 EGID, Lille, France
4. Univ. Lille, CNRS, Inserm, CHU Lille, Institut Pasteur de Lille, U1019-UMR 9017-CIIL-Center for Infection and Immunity of Lille, Lille, France.
5. VIB Center for Brain & Disease Research, KU Leuven, Leuven, Belgium.
6. Department of Neurosciences, Leuven Brain Institute, KU Leuven, Leuven, Belgium.
7. Univ. Lille, CNRS, Inserm, CHU Lille, Institut Pasteur de Lille, US41-UMS2014-PLBS, Lille, France.
8. Centre for Medical Genetics, Ghent University Hospital, Ghent, Belgium

^#^co-first

^##^co-last

*corresponding authors

Correspondence should be addressed to:

Marcos Costa, MD, PhD

INSERM UMR1167

Institut Pasteur de Lille

1 rue du Pr. Calmette

59019 Lille cedex, France

Tel: 00 33 (0)3 20 87 77 10

[marcos.costa@pasteur-lille.fr](mailto:marcos.costa@pasteur-lille.fr)

Pierre Dourlen, PhD

INSERM UMR1167

Institut Pasteur de Lille

1 rue du Pr. Calmette

59019 Lille cedex, France

Tel : 00 33 (0)3 20 87 77 10

[pierre.dourlen@pasteur-lille.fr](mailto:pierre.dourlen@pasteur-lille.fr)

# Generation and validation of transgenic drosophila lines expressing human BIN1 isoforms

To assess the role of BIN1 isoforms, we generated 3 transgenic Drosophila lines expressing 3 representative BIN1 isoforms, brain BIN1iso1, muscular BIN1iso8 and ubiquituous BIN1iso9 (supplementary method below). As a control, we also generated transgenic Drosophila lines expressing the longest dAmph isoform, dAmphA. We used the UAS/Gal4 system [1](Supplementary Fig. 1a) and ΦC31-mediated targeted insertion of transgenes to express identical levels of BIN1 isoforms [3]. We used the attP40 and attP2 landing sites on the second and third chromosomes. We got 1 to 5 lines for each construct resulting from independent identical insertion events. To test the expression of human BIN1 isoforms, we crossed the lines with an eye-specific GMR driver line and performed a western blot analysis. The BIN1 isoforms were expressed with an expected difference in the molecular weight (BIN1iso1 theoretical MW 65kDa > BIN1iso8 theoretical MW 50kDa > BIN1iso9 theoretical MW 48kDa). Surprisingly, some of the supposed-to-be identical lines expressed different levels of the same isoform. We selected 2 lines of each isoform inserted on the third chromosome and tested their expression at the RNA level by RT-qPCR (Supplementary method below, Supplementary Fig. 1b). We observed that basal expression levels were the same for all isoforms (BIN1iso1#3, BIN1iso8#1, BIN1iso8#2, BIN1iso9#2) except for two lines (BIN1iso1#1, BIN1iso9#1) expressing around twice as much BIN1 isoforms. This is likely due to the insertion of 2 copies of the transgenes. We repeated the western blot analysis for these lines which confirmed the higher expression of the two lines at the protein levels (Supplementary Fig. 1c). We decided to keep and use these lines to test for dose-dependent effects. We also observed that the basal level-expressing BIN1iso1 line exhibited non-significant higher protein levels of BIN1iso1 than BIN1iso8 and BIN1iso9 (significant difference only between BIN1iso1#3 and BIN1iso8#1, p=0.023, ANOVA with post-hoc Tukey test). This indicates that BIN1iso1 tended to be more stable than BIN1iso8 and BIN1iso9 when expressed in the Drosophila eye. In the article, if not specified, we have used the basal level-expressing lines (BIN1iso1#3, BIN1iso8#1, BIN1iso9#2) on the third chromosome and “BIN1iso1 high” or “BIN1iso9 high” refers to the BIN1iso1#1 and BIN1iso9#1 highly expressing lines.

# Characterization of dAmph^MI08903-TG4.0^ allele

The dAmph^MI08903-TG4.0^ allele contains a Trojan Gal4 exon cassette in the first intron of dAmph [2, 4] (Supplementary Fig. 2). This allows Gal4 expression under the control of dAmph endogenous promoter while arresting dAmph transcription thanks to a polyadenylation signal located 3’ of the GAL4. Because the first exon only contains 68nt of the dAmph coding DNA sequence, the expressed truncated protein consists only in around the first 25 amino acids of the protein and the mutation is likely null. To test this, we assessed dAmph protein expression and the climbing ability of compound heterozygous dAmph^MI08903-TG4.0^ flies with the known dAmph^5E3^ null allele [5]. We did not detect any dAmph in dAmph^MI08903-TG4.0/5E3^ compound heterozygous fly protein extract (Fig. 1b). In addition, these flies had strong locomotor defect. They had a climbing score close to the one of Amph^5E3/5E3^, around 2, whereas control flies had a climbing score close to 5 (Fig. 1C). These results indicate that the Amph^MI08903-TG4.0^ allele can be considered as a null allele.

# Supplementary methods

## Construction of Drosophila transgenesis vectors

BIN1iso1 cDNA, BIN1iso8 cDNA (kind gifts of J Laporte) and BIN1iso9 cDNAs (SC128163, OriGene Technologies, Inc., USA) were amplified by PCR using the forward primer CAAAATGGCAGAGATGGGCAGTAA and the reverse primer CTCGAGTCATGGGACCCTCTCAGTG, which allowed the insertion of a CAAA Kozak sequence upstream of the ATG and a CTCGAG XhoI restriction enzyme site after the stop codon. Of note, BIN1iso9 cDNA has two synonymous mutations, a C instead of a T at position 486 and a T instead of a C at position 864 from the initial ATG. Similarly dAmphA cDNA (LD19810 from the Drosophila Genomics Resource Center, Indiana University) was amplified by PCR using the forward primer CAAAATGACCGAAAATAAAGGCATAA and the reverse primer ACTTCACGCGTCCCATCTGACTCGAG. The PCR product was cloned into a pGEM-T Easy Vector (Promega). After sequence checking, we subcloned the insert using EcoRI and XhoI restriction enzyme into a pUAST-attB transgenesis vector. After sequence checking, transgenesis plasmids were sent to the Bestgene company (BestGene Inc, Chino Hills, USA) for embryo injection in y1 w67c23; P{CaryP}attP40 and y^1^ w^67c23^;; P{CaryP}attP2 lines (attP sites on the II and III chromosomes respectively).

For BIN1iso1 ΔEx7 and BIN1iso1 ΔCLAP, cDNAs were synthetized by Invitrogen GeneArt gene synthesis adding upstream an EcoRI restriction enzyme site from the pGEMT-easy and the CAAA Kozak sequence, and adding downstream an XhoI site to have identical sequences around the cDNA and to allow the comparison with BIN1iso1 transgenic flies. BIN1iso1 ΔEx7 and BIN1iso1 ΔCLAP cDNAs correspond to BIN1iso1 cDNA without Exon7 for the former and Exon13-14-15-16 for the latter. cDNA was subcloned into a pUAST attB transgenesis vector and transgenic flies were generated as described above.

## RT-qPCR

RNA was extracted from 30 Drosophila heads per condition using Trizol reagent (15596018, Invitrogen) according to manufacturer instructions. Retrotranscription was performed using ThermoScript™ RT-PCR System (11146-024, Invitrogen) and PCR was performed in the AriaMx Real-time PCR System (G8830A, Agilent Technologies). The following primers were used: for BIN1, forward primer ATGTTCAAGGTACAGGCCCA and reverse primer TCAGTGAAGTTCTCGGGGAA, both primers are localized in exons common to all isoforms corresponding to the SH3 domain; for RpL32, forward primer CCAAGGACTTCATCCGCCACC and reverse primer GCGGGTGCGCTTGTTCGATCC.

# References

1. Brand AH, Perrimon N (1993) Targeted gene expression as a means of altering cell fates and generating dominant phenotypes. Development 118:401–15

2. Diao F et al. (2015) Plug-and-play genetic access to drosophila cell types using exchangeable exon cassettes. Cell Rep 10:1410–21. doi: 10.1016/j.celrep.2015.01.059

3. Groth AC, Fish M, Nusse R, Calos MP (2004) Construction of Transgenic Drosophila by Using the Site-Specific Integrase from Phage φC31. Genetics 166:1775–1782. doi: 10.1534/genetics.166.4.1775

4. Lee PT et al. (2018) A gene-specific T2A-GAL4 library for drosophila. Elife 7. doi: 10.7554/eLife.35574

5. Leventis PA et al. (2001) Drosophila Amphiphysin is a post-synaptic protein required for normal locomotion but not endocytosis. Traffic 2:839–50
