# Supplementary material for: The Alzheimer susceptibility gene BIN1 induces isoform-dependent neurotoxicity through early endosome defects
Source: Acta Neuropathol Commun. 2022 Jan 8;10:4. doi: 10.1186/s40478-021-01285-5 (PMC8742943; doi:10.1186/s40478-021-01285-5)

Supplementary Fig. 1: **Creation of transgenic lines expressing human BIN1 isoforms**. **a** Scheme of the Gal4/UAS system with the example of a GMR driver and a UAS construct expressing BIN1. **b** RT-qPCR analysis of BIN1 isoforms for 2 lines of each isoforms on the third chromosome (ANOVA, post-hoc Tukey, * p<0.05, **** p<0.0001). **c** Western blot analysis of BIN1 isoforms for the same lines. Tubulin is used as a loading control (ANOVA, post-hoc Tukey, * p<0.05, ** p<0.01, **** p<0.0001).

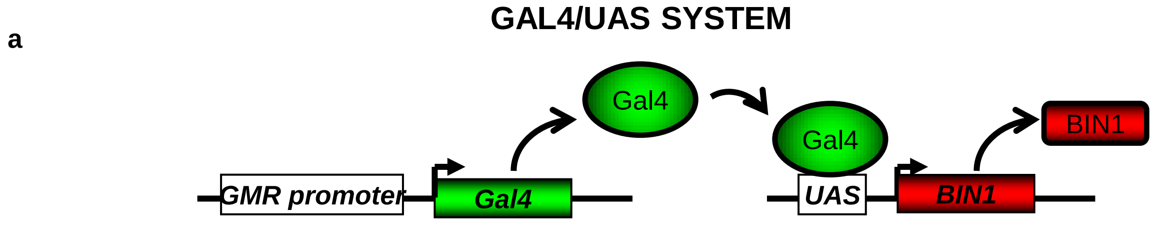

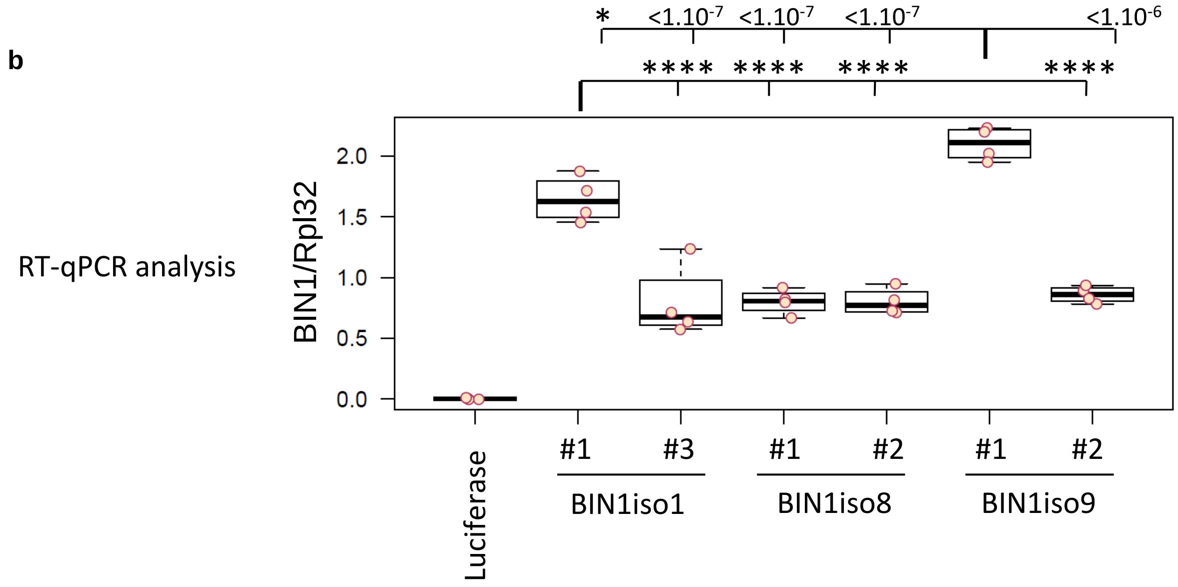

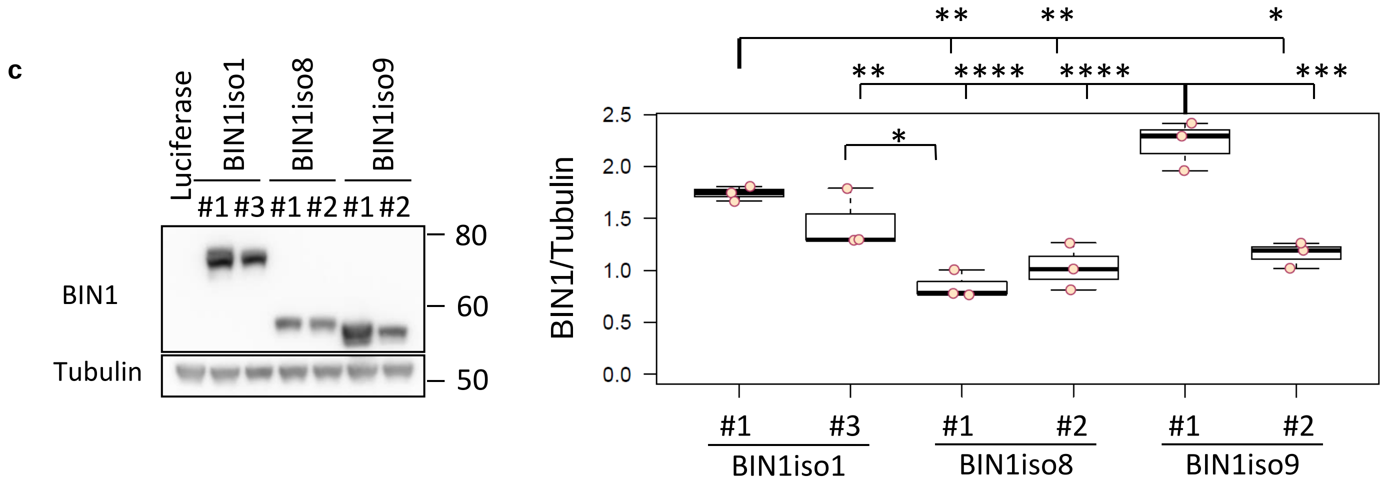

Supplement: Supplementary file 2 — Additional file 2. Figure S1. Creation of transgenic lines expressing human BIN1 isoforms. [file 40478_2021_1285_MOESM2_ESM.pdf]
