# Supplementary material for: The Alzheimer susceptibility gene BIN1 induces isoform-dependent neurotoxicity through early endosome defects
Source: Acta Neuropathol Commun. 2022 Jan 8;10:4. doi: 10.1186/s40478-021-01285-5 (PMC8742943; doi:10.1186/s40478-021-01285-5)

Supplementary Fig. 2: Scheme of Amph$^{MI08903\text{-}TG4.0}$ allele. SA=Splice acceptor site, SD=Splice donnor site.

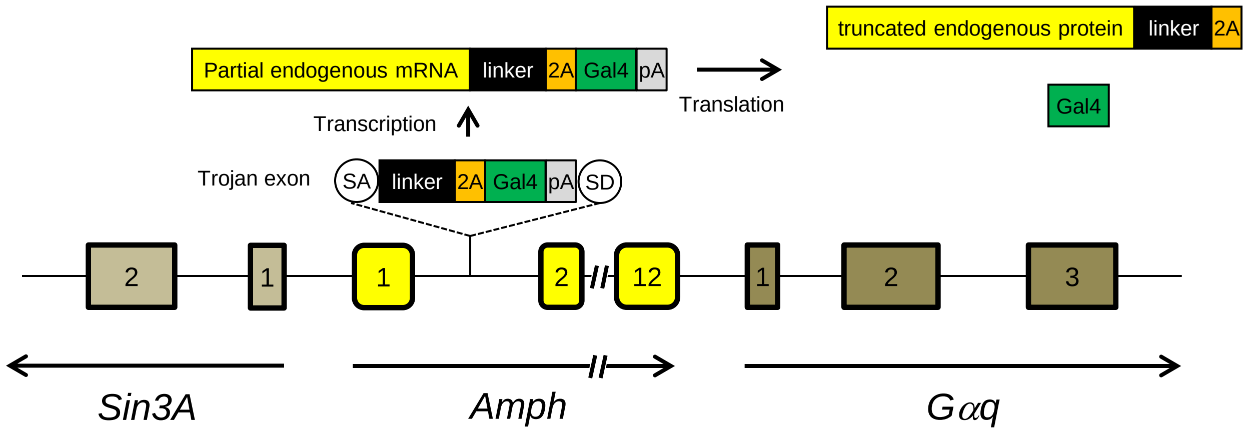

Supplement: Supplementary file 3 — Additional file 3. Figure S2. Scheme of AmphMI08903-TG4.0 allele. [file 40478_2021_1285_MOESM3_ESM.pdf]
