# Supplementary material for: The Alzheimer susceptibility gene BIN1 induces isoform-dependent neurotoxicity through early endosome defects
Source: Acta Neuropathol Commun. 2022 Jan 8;10:4. doi: 10.1186/s40478-021-01285-5 (PMC8742943; doi:10.1186/s40478-021-01285-5)

Supplementary Fig. 3 : Generation and test of transgenic Drosophila expressing truncated human BIN1-1 forms for the Exon7 (BIN1-1 ΔEx7) and the CLAP domain (BIN1-1 ΔCLAP). The two UAS constructs were inserted in the attP40 (2nd chromosome) and the attP2 (3rd chromosome) landing sites thanks to the ΦC31 integrase. We obtained 3 to 5 independent lines per landing sites. **a** Western blot analysis of the transgene expression under rh1 driver and quantification (**b**). Each line expressed a BIN1 form with the expected molecular weight (BIN1-1 > BIN1-1 ΔEx7 > BIN1-1 ΔCLAP > BIN1-9). Expression levels were similar between lines with a non-significant tendency of decreased levels for BIN1-1 ΔCLAP. Two lines of each category were tested for their ability to induce photoreceptor neuron degeneration. **c** Quantification of the truncated BIN1-1 form-induced neurodegeneration. Results were identical whatever the insertion sites and independent lines. Loss of Exon7 partially rescued photoreceptor neurons whereas loss of the CLAP nearly totally rescued them. We can wonder if the reduced expression of the BIN1-1 ΔCLAP contribute to its lesser toxicity but we have seen with BIN1-1 and BIN1-9 that the effect is likely dose-independent. Results of BIN1-1 ΔEx7 attP2 #1 and of BIN1-1 ΔCLAP attP2 #1 were used in Figure 2F and 2G.

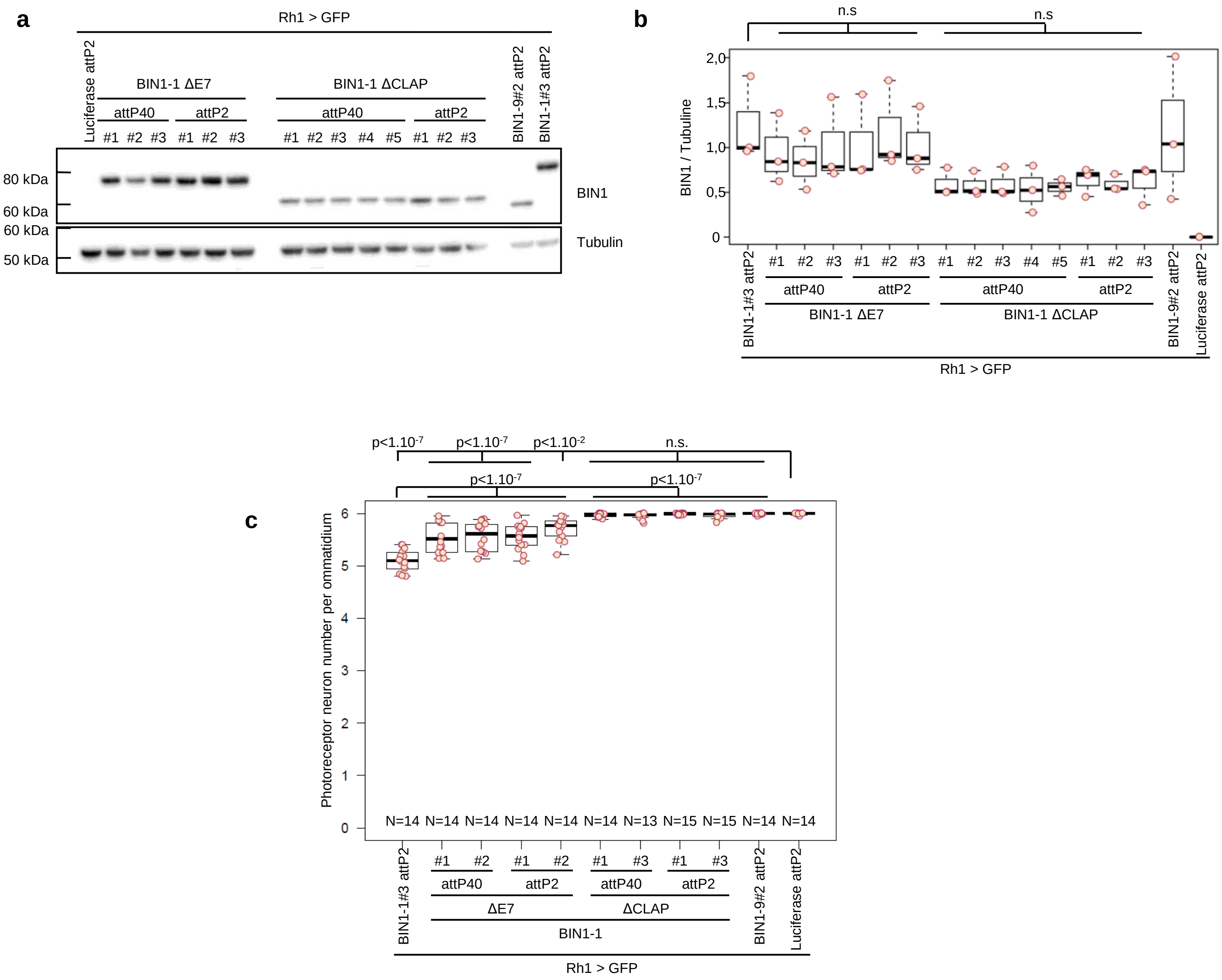

Supplement: Supplementary file 4 — Additional file 4. Figure S3. Generation and test of transgenic Drosophila expressing truncated human BIN1-1 forms for the Exon7 (BIN1-1 ΔEx7) and the CLAP domain (BIN1-1 ΔCLAP). [file 40478_2021_1285_MOESM4_ESM.pdf]
