# Supplementary material for: The Alzheimer susceptibility gene BIN1 induces isoform-dependent neurotoxicity through early endosome defects
Source: Acta Neuropathol Commun. 2022 Jan 8;10:4. doi: 10.1186/s40478-021-01285-5 (PMC8742943; doi:10.1186/s40478-021-01285-5)

Supplementary Fig. 4 : Electron microscopy images of BIN1-1-induced photoreceptor degeneration in Drosophila eyes. a View of several ommatidia expressing luciferase as a control (left) or BIN1-1 (right) in 15 day-old flies. BIN1-1 expressing ommatidia exhibit many vesicles. **b** Longitudinal view of photoreceptor neurons in the distal part of the retina. Under the cornea (¤) and pseudocone (*), a photoreceptor neuron is filled with vesicles (arrow). The nucleus (arrow in the first inset) is squeezed but the chromatine seem normal. The cytoplasm is pushed on the side against the plasma membrane, which is not disrupted (arrow in the second inset). **c** Image showing a multilamellar body.

a

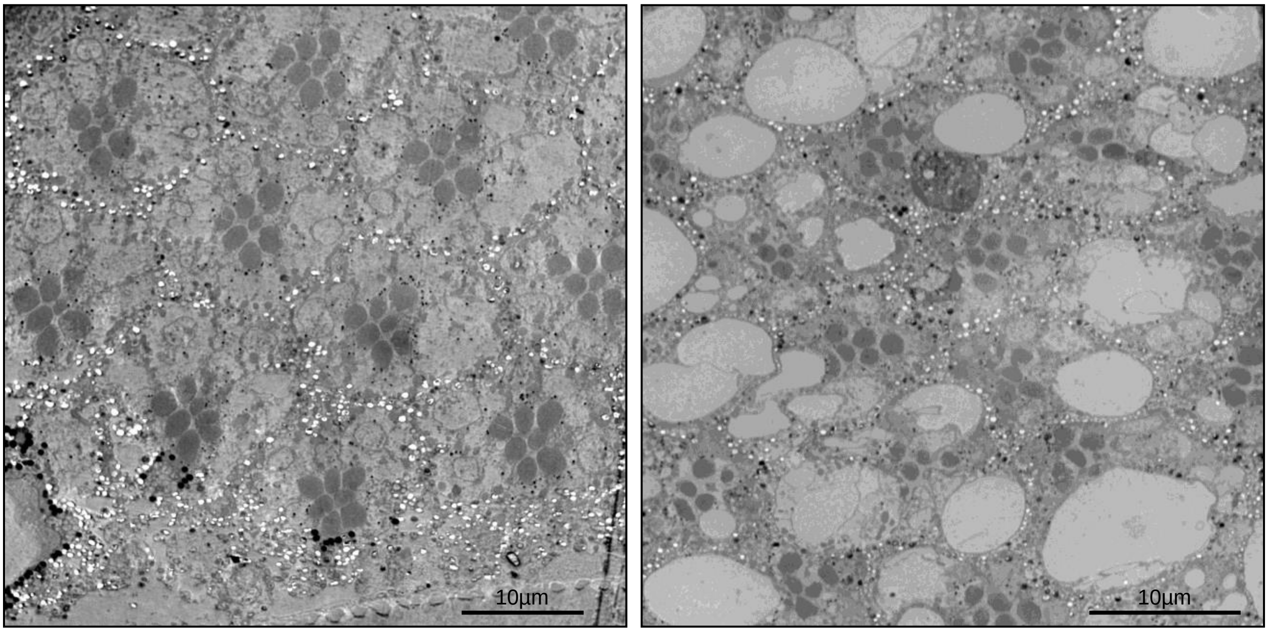

b

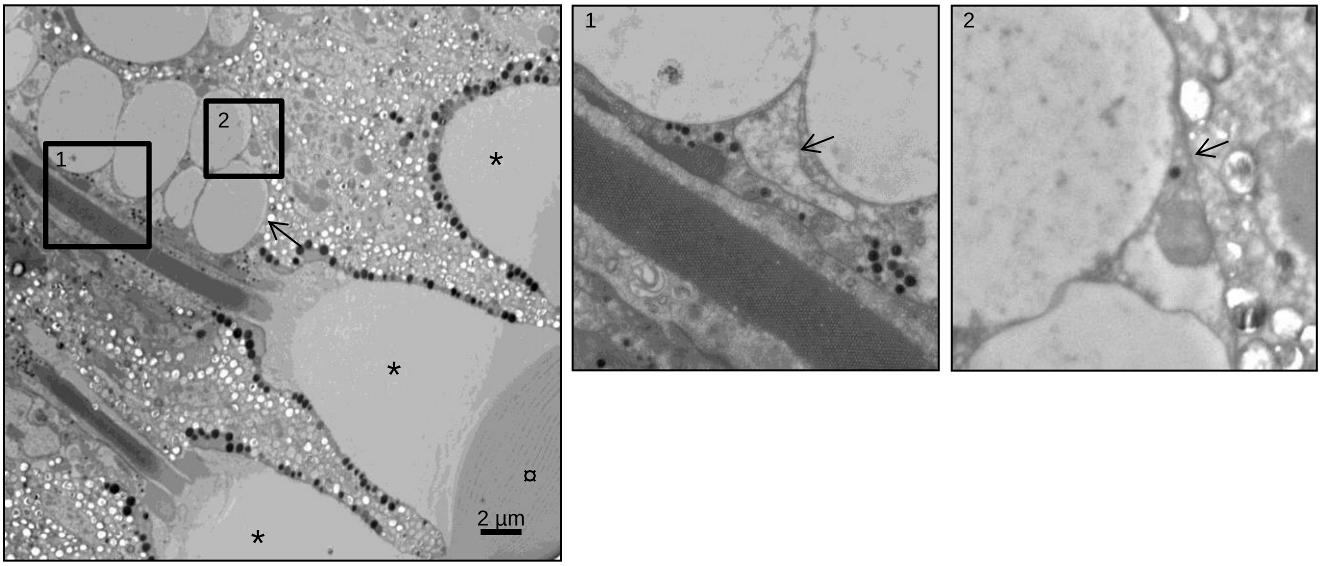

c

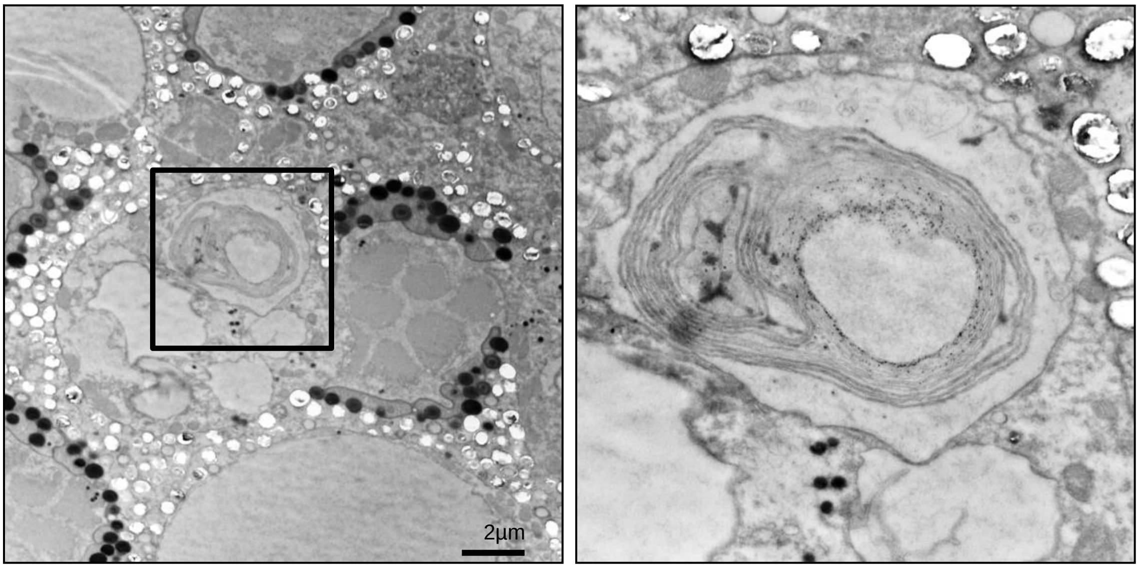

Supplementary Fig. 4 : Electron microscopy images of BIN1-1-induced photoreceptor degeneration in Drosophila eyes. d BIN1-1 expressing retina exhibited dying photoreceptor neurons. They first started to round up, their cytoplasm became electron-dense with abnormal mitochondria (arrow, upper left panel). They shrank (arrow, upper right panel) and were finally phagocytosed by the adjacent interommatidial cell (arrow, lower left panel).

d

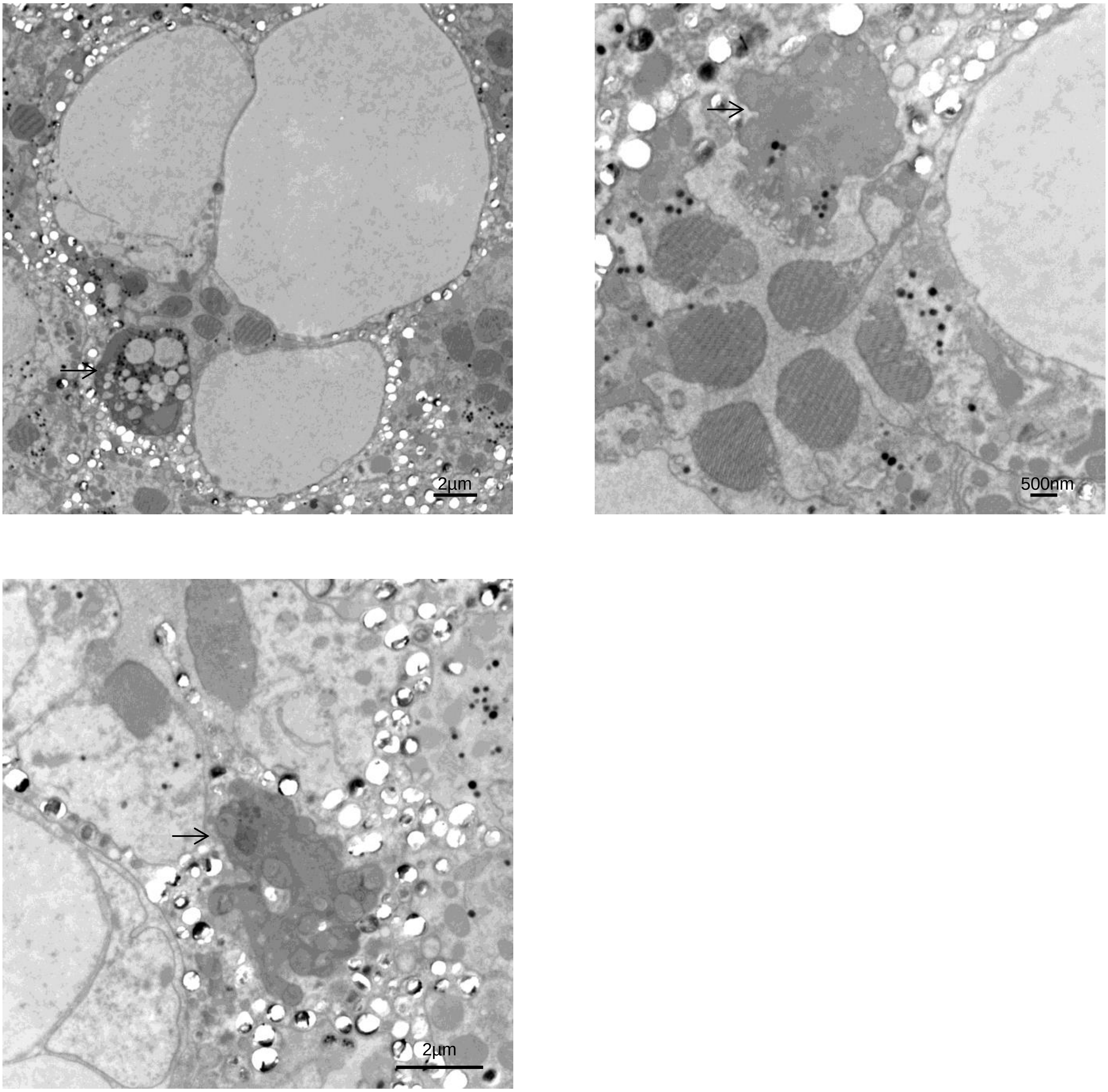

Supplement: Supplementary file 5 — Additional file 5. Figure S4. Electron microscopy images of BIN1-1-induced photoreceptor degeneration in Drosophila eyes. [file 40478_2021_1285_MOESM5_ESM.pdf]
