# Supplementary material for: The Alzheimer susceptibility gene BIN1 induces isoform-dependent neurotoxicity through early endosome defects
Source: Acta Neuropathol Commun. 2022 Jan 8;10:4. doi: 10.1186/s40478-021-01285-5 (PMC8742943; doi:10.1186/s40478-021-01285-5)

Supplementary Fig. 5: Screening of organelle markers in BIN1-1-induced degenerating photoreceptor neurons. BIN1-1-expressing flies were crossed with line expressing green fluorescent marker for ER **a**, Golgi **b**, plasma membrane **c**, early endosome **d**, late endosome/multivesicular body **e**, recycling endosome **f**, lysosome **g** and autophagosome **h**, and we let flies age for 1 and 2 weeks before dissection and immunofluorescence. Rh1 and actin labelling (respectively white and red in merge images) were used to visualize retinal structure and only green channels and merge images are shown. Note that the KDEL:GFP marker labelled nuclear envelop (arrows in **A**), early and late endosome/multivesicular body marker labelled small to middle size vesicles (arrows in **d**, **e**), the evi:GFP marker also labelled bigger vesicles (see image of one week-old flies) and the interrhabdomeric space (arrowhead in **e**), which likely corresponds to released exosomes, the Lamp2:GFP marker labelled on rare cases some middle to big size vesicles (arrow in **g**) and the autophagosome marker GFP:LC3 labelled small structures in the control and BIN1-1 conditions (arrows in **h**).

a ER

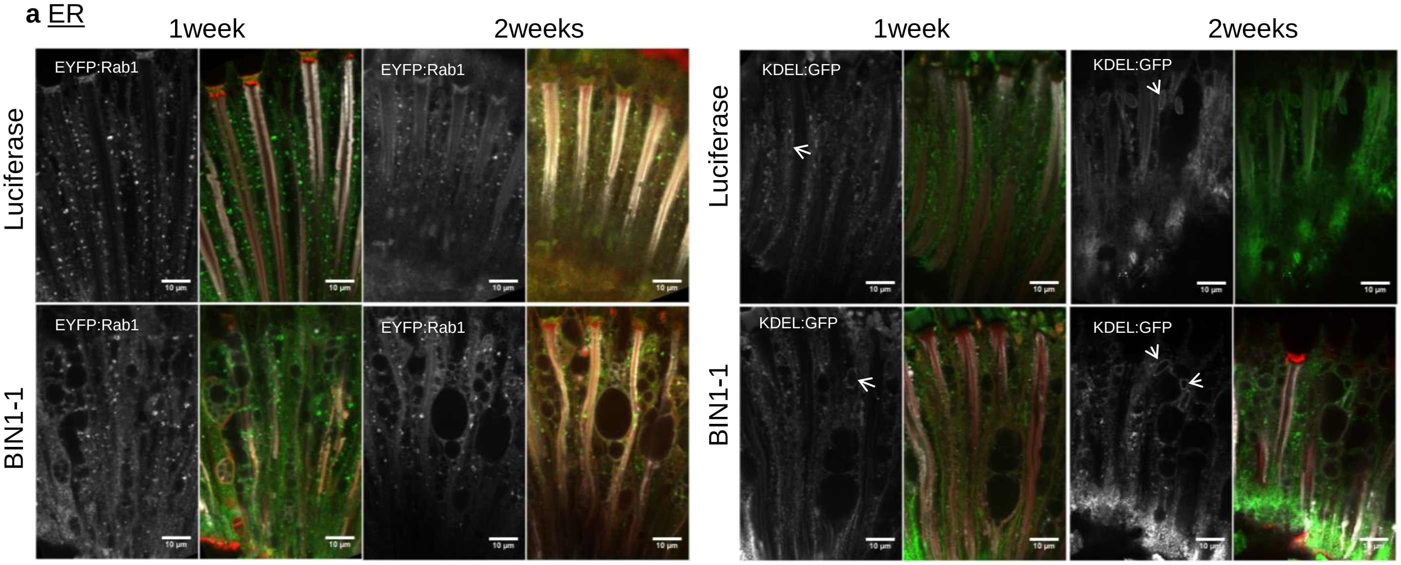

b Golgi

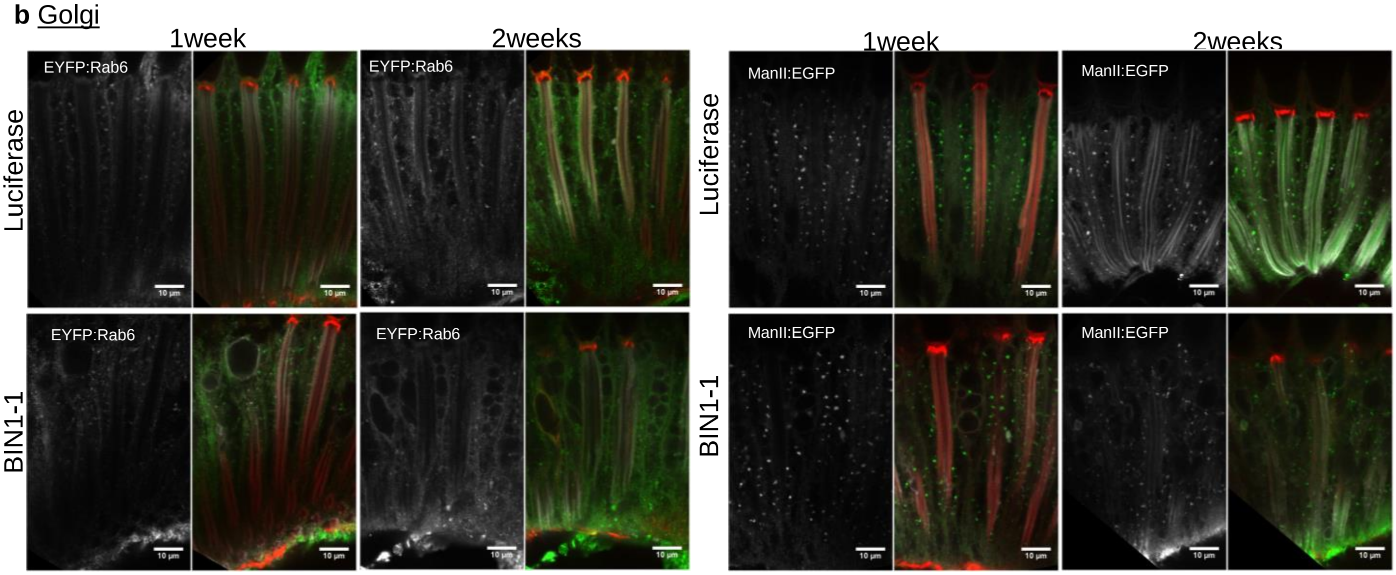

c Plasma membrane

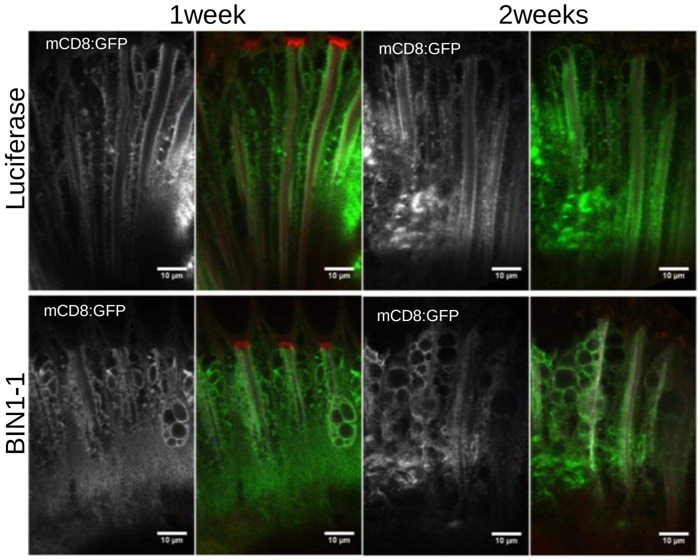

Supplementary Fig. 5: Screening of organelle markers in BIN1-1-induced degenerating photoreceptor neurons. BIN1-1-expressing flies were crossed with line expressing green fluorescent marker for ER **a**, Golgi **b**, plasma membrane **c**, early endosome **d**, late endosome/multivesicular body **e**, recycling endosome **f**, lysosome **g** and autophagosome **h**, and we let flies age for 1 and 2 weeks before dissection and immunofluorescence. Rh1 and actin labelling (respectively white and red in merge images) were used to visualize retinal structure and only green channels and merge images are shown. Note that the KDEL:GFP marker labelled nuclear envelop (arrows in **A**), early and late endosome/multivesicular body marker labelled small to middle size vesicles (arrows in **d**, **e**), the evi:GFP marker also labelled bigger vesicles (see image of one week-old flies) and the interrhabdomeric space (arrowhead in **e**), which likely corresponds to released exosomes, the Lamp2:GFP marker labelled on rare cases some middle to big size vesicles (arrow in **g**) and the autophagosome marker GFP:LC3 labelled small structures in the control and BIN1-1 conditions (arrows in **h**).

d Early endosome

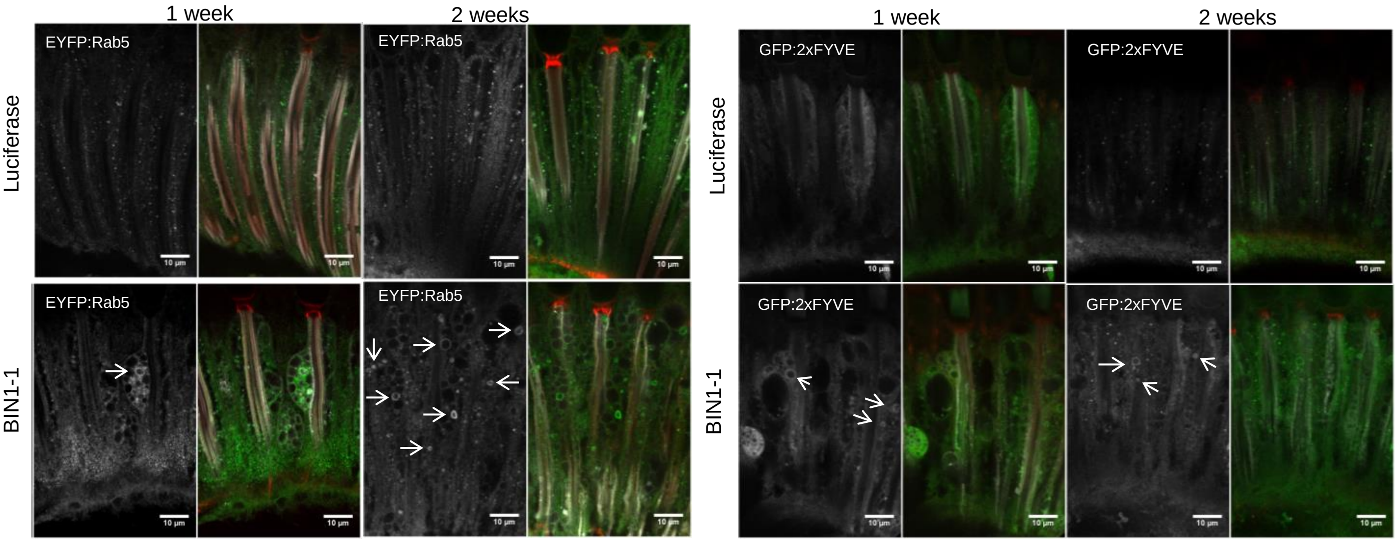

e Late endosome

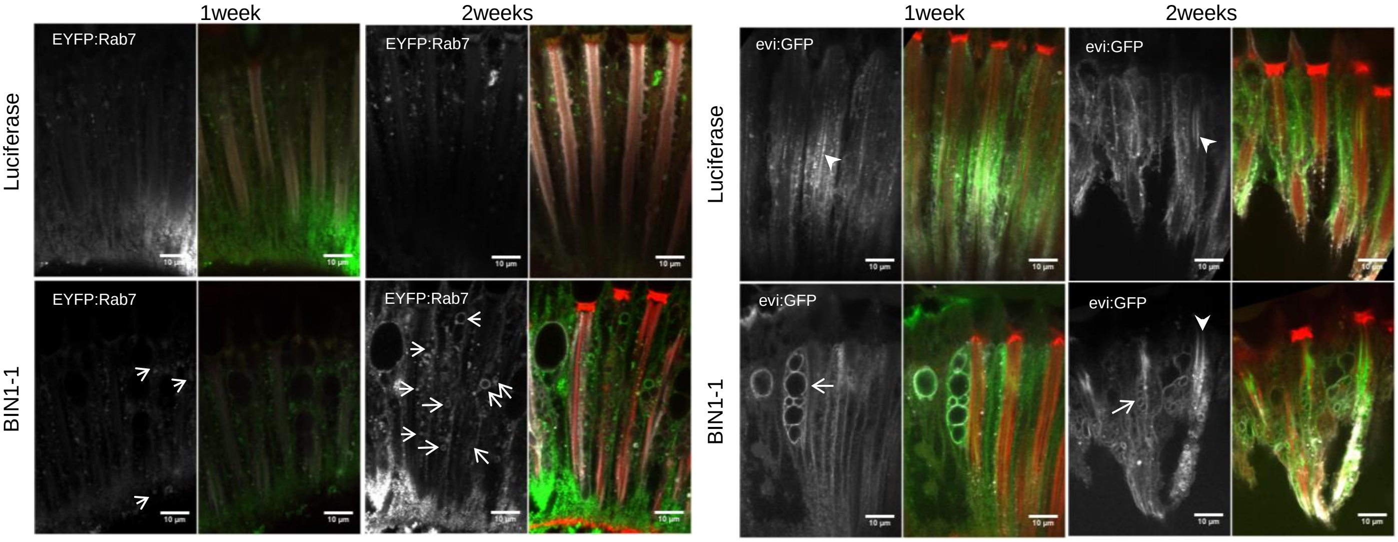

f Recycling endosome

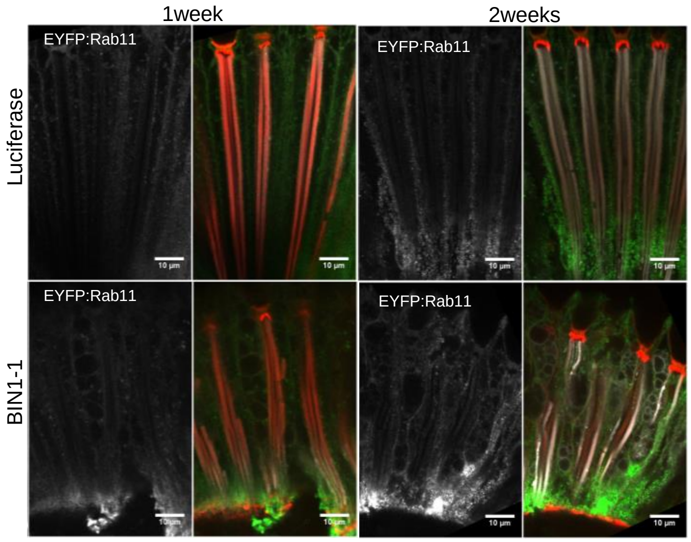

Supplementary Fig. 5: Screening of organelle markers in BIN1-1-induced degenerating photoreceptor neurons. BIN1-1-expressing flies were crossed with line expressing green fluorescent marker for ER **a**, Golgi **b**, plasma membrane **c**, early endosome **d**, late endosome/multivesicular body **e**, recycling endosome **f**, lysosome **g** and autophagosome **h**, and we let flies age for 1 and 2 weeks before dissection and immunofluorescence. Rh1 and actin labelling (respectively white and red in merge images) were used to visualize retinal structure and only green channels and merge images are shown. Note that the KDEL:GFP marker labelled nuclear envelop (arrows in **A**), early and late endosome/multivesicular body marker labelled small to middle size vesicles (arrows in **d**, **e**), the evi:GFP marker also labelled bigger vesicles (see image of one week-old flies) and the interrhabdomeric space (arrowhead in **e**), which likely corresponds to released exosomes, the Lamp2:GFP marker labelled on rare cases some middle to big size vesicles (arrow in **g**) and the autophagosome marker GFP:LC3 labelled small structures in the control and BIN1-1 conditions (arrows in **h**).

g Lysosome

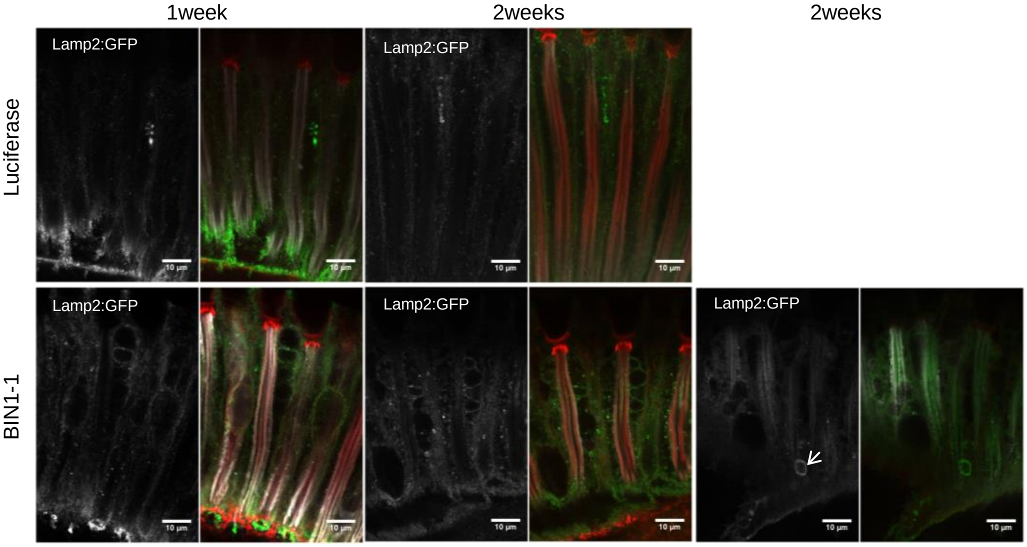

h autophagosome

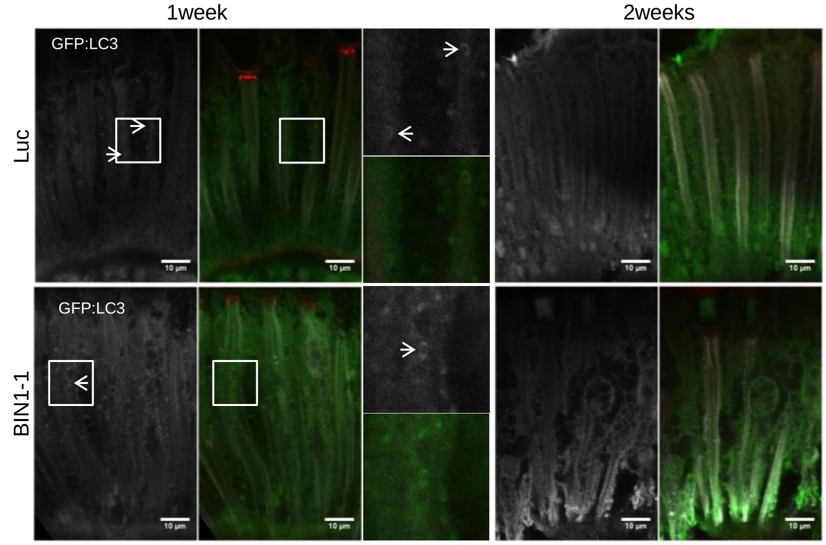

Supplement: Supplementary file 6 — Additional file 6. Figure S5. Screening of organelle markers in BIN1-1-induced degenerating photoreceptor neurons. [file 40478_2021_1285_MOESM6_ESM.pdf]
