# Supplementary material for: The Alzheimer susceptibility gene BIN1 induces isoform-dependent neurotoxicity through early endosome defects
Source: Acta Neuropathol Commun. 2022 Jan 8;10:4. doi: 10.1186/s40478-021-01285-5 (PMC8742943; doi:10.1186/s40478-021-01285-5)

Supplementary Fig. 6: Effect of Rab4 modulation on BIN1iso1 neurotoxicity in Drosophila photoreceptor neurons. Quantification of the BIN1iso1-induced neurodegeneration upon overexpression of Rab4:mRFP, YFP:Rab4, YFP:Rab4^{S22N} (DN) and YFP:Rab4^{Q67L} (CA). The number of photoreceptor neurons per ommatidia is quantified in 15-day-old flies. Only the Rab4:mRFP condition is significantly different than the associated control mCD8:mRFP. Statistical analysis was performed using a Kruskal Wallis test (p=0.02236) followed by Mann Whitney comparison (** p<0.01).

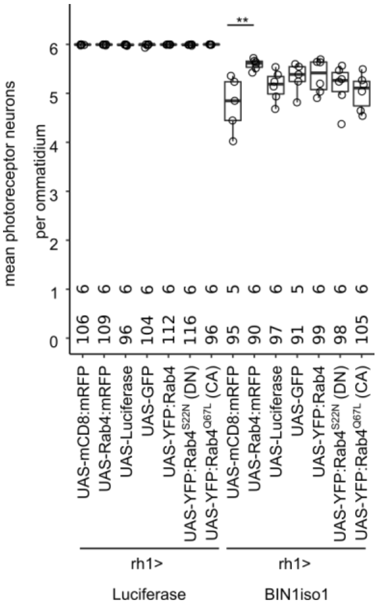

Supplement: Supplementary file 7 — Additional file 7. Figure S6. Effect of Rab4 modulation on BIN1iso1 neurotoxicity in Drosophila photoreceptor neurons. [file 40478_2021_1285_MOESM7_ESM.pdf]
