# Supplementary material for: The Alzheimer susceptibility gene BIN1 induces isoform-dependent neurotoxicity through early endosome defects
Source: Acta Neuropathol Commun. 2022 Jan 8;10:4. doi: 10.1186/s40478-021-01285-5 (PMC8742943; doi:10.1186/s40478-021-01285-5)

Supplementary Fig. 7. Graphic representation of proportions of cell types (in percentages) in *BIN1 KO* and *WT* cerebral organoids. 190-day old organoids were subjected to single nucleus RNA sequencing. Specific cell type populations within the organoids are indicated by different colours.

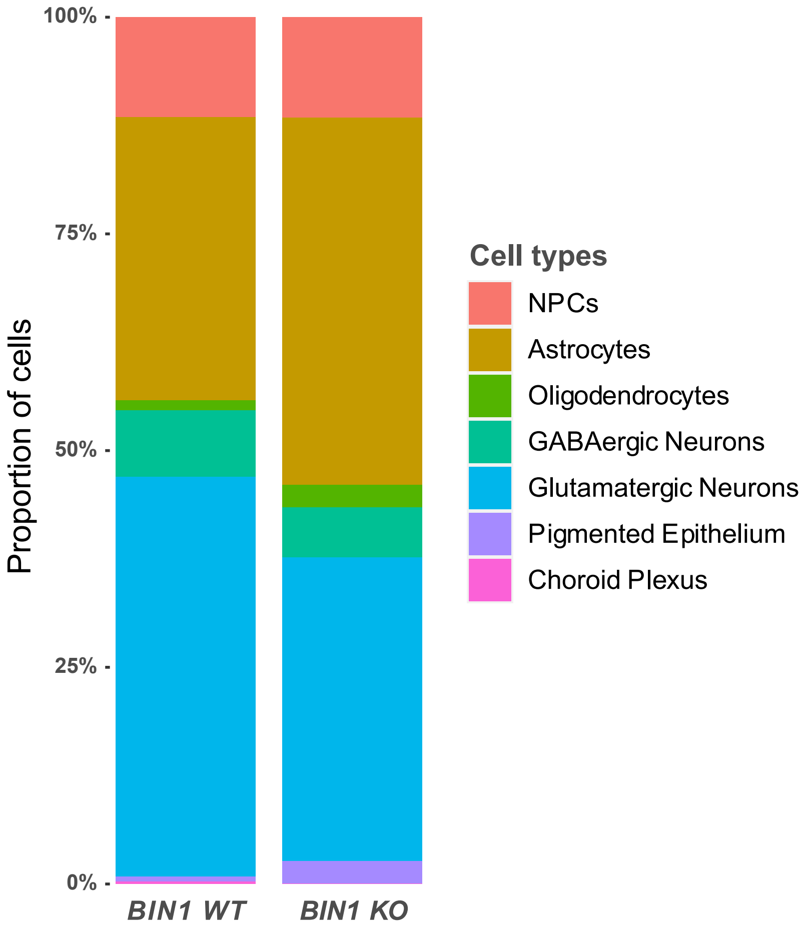

Supplement: Supplementary file 8 — Additional file 8. Figure S7. Graphic representation of proportions of cell types (in percentages) in BIN1 KO and WT cerebral organoids. [file 40478_2021_1285_MOESM8_ESM.pdf]
