# Supplementary material for: The Alzheimer susceptibility gene BIN1 induces isoform-dependent neurotoxicity through early endosome defects
Source: Acta Neuropathol Commun. 2022 Jan 8;10:4. doi: 10.1186/s40478-021-01285-5 (PMC8742943; doi:10.1186/s40478-021-01285-5)

Supplementary Fig. 8. Decreased APP/β-CTF levels in *BIN1* KO cerebral organoids. (a) Immunoblot for APP C-terminal and ACTIN in 190-day-old *BIN1* WT and KO cerebral organoids. Bands corresponding to the full-length product (110 kDa) and the C-terminal fragment (10 kDa) are indicated. **(b)** Graphs showing the quantifications of the ratios: β-CTF/β-ACTIN, β-CTF/full-length APP, and full-length APP/β-ACTIN normalized to WT (*p=0.0177; **p=0.0034; Unpaired t-test; N = 5 organoids/genotype). **(c)** Immunoblot similar to (a) for 6-week-old 2D cell cultures. **(d)** Quantifications of the ratios: β-CTF/β-ACTIN, β-CTF/full-length APP, and full-length APP/β-ACTIN (#p=0.0867; Unpaired t-test; N=4 independent cell cultures).

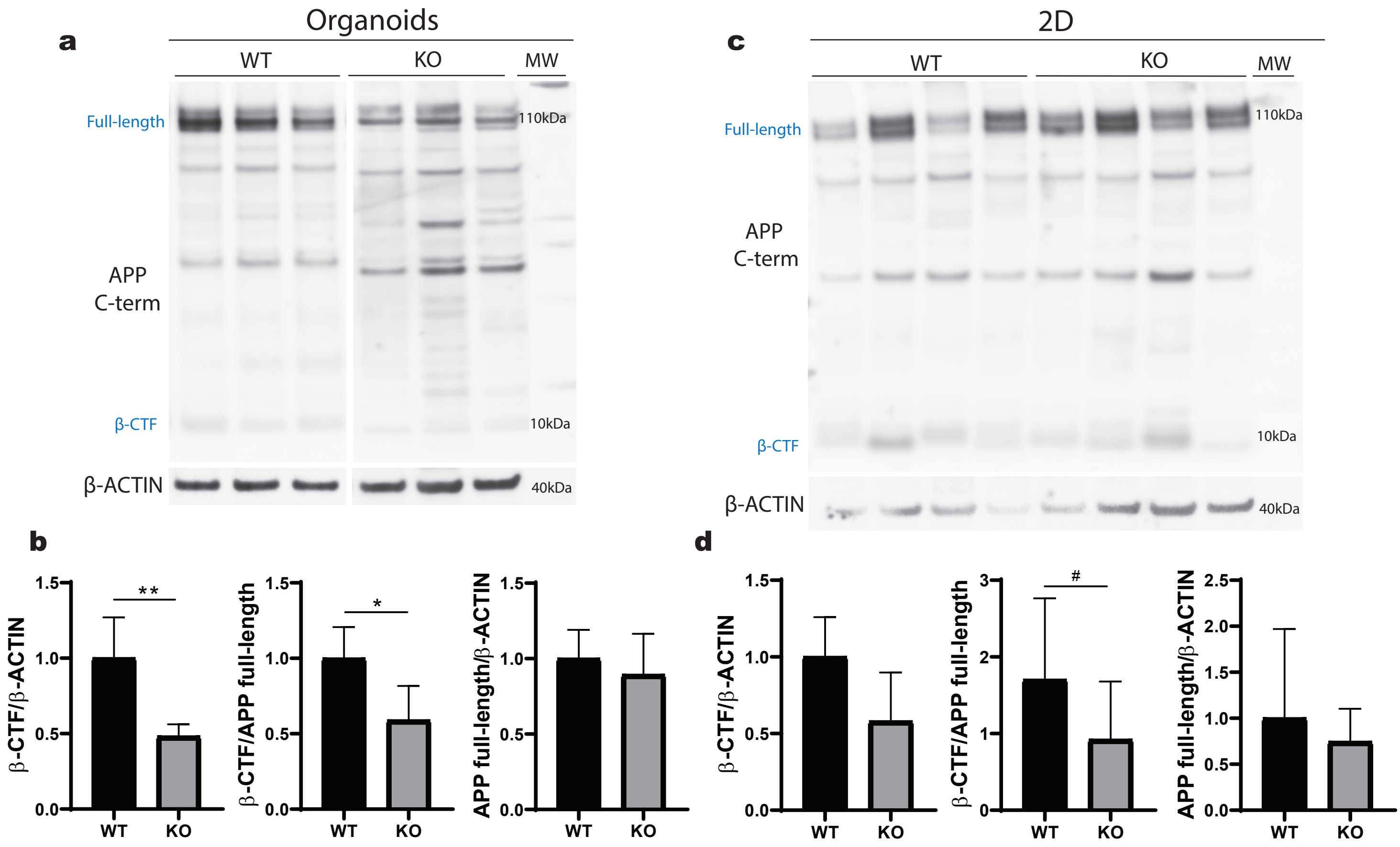

Supplement: Supplementary file 11 — Additional file 11. Figure S8. Decreased APP/β-CTF levels in BIN1 KO cerebral organoids. [file 40478_2021_1285_MOESM11_ESM.pdf]
